# Milk- and solid-feeding practices and daycare attendance are associated with differences in bacterial diversity, predominant communities, and metabolic and immune function of the infant gut microbiome

**DOI:** 10.3389/fcimb.2015.00003

**Published:** 2015-02-05

**Authors:** Amanda L. Thompson, Andrea Monteagudo-Mera, Maria B. Cadenas, Michelle L. Lampl, M. A. Azcarate-Peril

**Affiliations:** ^1^Department of Anthropology, University of North CarolinaChapel Hill, NC, USA; ^2^Microbiome Core Facility, Center for Gastrointestinal Biology and Disease, University of North CarolinaChapel Hill, NC, USA; ^3^Department of Anthropology and Center for the Study of Human Health, Emory UniversityAtlanta, GA, USA; ^4^Department of Cell Biology and Physiology, School of Medicine, University of North CarolinaChapel Hill, NC, USA

**Keywords:** infant gut microbiome, breastfeeding, metagenomics, daycare, feeding transitions

## Abstract

The development of the infant intestinal microbiome in response to dietary and other exposures may shape long-term metabolic and immune function. We examined differences in the community structure and function of the intestinal microbiome between four feeding groups, exclusively breastfed infants before introduction of solid foods (EBF), non-exclusively breastfed infants before introduction of solid foods (non-EBF), EBF infants after introduction of solid foods (EBF+S), and non-EBF infants after introduction of solid foods (non-EBF+S), and tested whether out-of-home daycare attendance was associated with differences in relative abundance of gut bacteria. Bacterial 16S rRNA amplicon sequencing was performed on 49 stool samples collected longitudinally from a cohort of 9 infants (5 male, 4 female). PICRUSt metabolic inference analysis was used to identify metabolic impacts of feeding practices on the infant gut microbiome. Sequencing data identified significant differences across groups defined by feeding and daycare attendance. Non-EBF and daycare-attending infants had higher diversity and species richness than EBF and non-daycare attending infants. The gut microbiome of EBF infants showed increased proportions of *Bifidobacterium* and lower abundance of Bacteroidetes and Clostridiales than non-EBF infants. PICRUSt analysis indicated that introduction of solid foods had a marginal impact on the microbiome of EBF infants (24 enzymes overrepresented in EBF+S infants). In contrast, over 200 bacterial gene categories were overrepresented in non-EBF+S compared to non-EBF infants including several bacterial methyl-accepting chemotaxis proteins (MCP) involved in signal transduction. The identified differences between EBF and non-EBF infants suggest that breast milk may provide the gut microbiome with a greater plasticity (despite having a lower phylogenetic diversity) that eases the transition into solid foods.

## Introduction

During the first year of life the human infant gut microbiome undergoes rapid maturation, changing dynamically in response to early environmental exposures, such as delivery type, hygiene measures, and diet (Guarner and Malagelada, 2003). Infant feeding practices, particularly breast- and formula-feeding, have been shown to influence the structure and function of this developing microbiome. Human milk provides the infant with a rich microbial consortium and a variety of oligosaccharides, prebiotics ensuring gut colonization by microbes beneficial for metabolism and immune development (Jeurink et al., 2013). Differential development of the intestinal microbiome in response to human milk may underlie documented differences in infectious illness morbidity, allergy, and obesity risk between breast- and formula-fed infants (Fanaro et al., 2003; Ip et al., 2007; Donnet-Hughes et al., 2010).

The gut microbiota of breastfed infants has been characterized as having less diverse colonization and greater proportions of *Bifidobacterium* and *Lactobacillus* species compared to non-breastfed infants. A recent study by Tannock et al. (2013) identified Bifidobacteriaceae (61%), Enterobacteriaceae (8%), and Coriobacteriaceae (6%) as the most abundant bacterial families detected in stool from breastfed babies. The microbiome of formula-fed infants is more diverse with greater proportions of *Bacteroides*, *Clostridium* and enterobacteria (Orrhage and Nord, 1999; Vael and Desager, 2009; Marques et al., 2010). The presence and/or predominance of *Bifidobacterium* in exclusively breastfed compared to non-exclusively breastfed or formula-fed infants is still under debate. While numerous studies have reported a lower abundance of *Bifidobacterium* in formula-fed infants relative to breast-fed infants (Yoshioka et al., 1983; Favier et al., 2002; Hopkins et al., 2005), other studies have reported *Bifidobacterium* as the dominant taxa in both groups (Gomez-Llorente et al., 2013; Tannock et al., 2013; Yatsunenko et al., 2012). The predominant families in mixed-fed infants have been less well characterized after the neonatal period, but their microbiomes have been assumed to be intermediate between those of breast- and formula-fed infants (Orrhage and Nord, 1999).

The composition of the early gut microbiome changes in both breast- and formula-fed infants as solids foods are introduced into the diet (Guarner and Malagelada, 2003; Amarri et al., 2006; Nielsen et al., 2007; Palmer et al., 2007). The transition from a milk-based to a solids-based diet exposes infants to novel non-digestible plant carbohydrates, animal protein, and fats providing new substrates for the survival and dominance of bacterial species not supported by breastmilk and/or formula (Parrett and Edwards, 1997; Fallani et al., 2011). Introduction of solid foods has been associated with increased populations of *Bacteroides* (Koenig et al., 2011) and decreased populations of bifidobacteria, enterobacteria, and some *Clostridium* species (Fallani et al., 2011). In older children and adults, dietary composition, particularly the balance between carbohydrates and protein/animal fat, is associated with microbiome changes with greater *Bacteroides* abundance associated with diets higher in protein and animal fat and *Prevotella* abundance associated with greater carbohydrate intake relative to meat and dairy (De Filippo et al., 2010; Wu et al., 2011). Along with the increased abundance of *Bacteroides*, changes in bacterial populations caused by introduction of solid foods have been correlated with increased short chain fatty acids (SCFAs) levels (Koenig et al., 2011). Such diet-induced differences in the gut microbiota may contribute to host metabolism and immune function by regulating genes involved in lipid and carbohydrate metabolism, altering endocrine functions, increasing inflammatory responses, and influencing energy balance and body weight (Gill et al., 2006; Greiner and Backhed, 2011; Nauta et al., 2013). Yet, few studies have examined the effects of the solid feeding transition on the composition of the gut microbiota or its function longitudinally in a cohort of healthy, predominantly breastfed infants with well-documented diet, growth, and morbidity information.

To investigate the impact of infant feeding patterns (exclusive breastfeeding and mixed-feeding of breastmilk and formula) and the subsequent introduction of solid foods on the development and function of the gut microbiome, we analyzed the microbiome of stool samples (*N* = 49) collected longitudinally from 9 American infants, ages 2 weeks to 14 months, by 16S rRNA amplicon pyrosequencing. We described the species diversity associated with exclusive breastfeeding (EBF) and mixed breast and formula feeding (non-exclusively breast-fed, non-EBF) and tested whether the introduction of solid foods was associated with differences in bacterial composition in these groups. Additionally, we used PICRUSt (Langille et al., 2013) to construct a community-level metabolic network of the microbiome and compared the abundance of pathways across feeding groups to identify metabolic differences associated with feeding patterns and transitions.

As a secondary aim, we tested for significant differences in the gut microbiome by out-of-home daycare attendance. Young children in out-of-home daycare settings have higher risk of gastrointestinal and respiratory infections (Augustine et al., 2013). A number of recent trials have shown that probiotic supplementation reduces the incidence and duration of diarrheal episodes in children in out-of-home care, suggesting that this morbidity may be due to differences in the composition and function of the microbiome (Gutierrez-Castrellon et al., 2014). Thus, we examined whether daycare attendance was associated with differences in the microbiome beyond those attributable to feeding practices.

## Materials and methods

### Sample

Stool samples (*n* = 49) were collected from 9 infants (5 males, 4 females) participating in a prospective, mixed-longitudinal study investigating infant growth and development during the first year of life (Thompson and Lampl, 2013). As previously described, infants in the parent study were recruited from daycare centers, lactation support groups, and Atlanta-area universities. At recruitment, infants were aged 7 days to 10.5 months (median age at entry 19.6 weeks, IQR = 10.8–32.8 weeks) and were followed weekly for a median of 16 weeks (IQR = 16–20.8 weeks). All participating infants were born after 37 gestational weeks with birthweights >2500 g. Infants were chosen for the current analytic sample if they had a minimum of two retained stool samples with at least one preceding and one following a feeding transition, such as supplementation with formula or introduction of solid foods. Stool samples in the current study were from infants aged 13 days to 14 months of age.

Characteristics of infants in the analytic sample are described in Table 1. Eight of nine infants received breastmilk, four of nine infants were exclusively breastfed during at least one of the sample collection periods, and one infant received formula and solids with no breastmilk during the study period. Infants were breastfed for a median of 9 months (range: 5.2–13 months). Mixed fed infants were all predominantly breastfed, receiving the equivalent of one to two bottles of formula per day. Solid foods were introduced at a median age of 5 months (range: 4–6 months). Four of nine infants attended out-of-home daycare. Two of the nine infants in daycare and none of the five infants cared for in-home received a narrow spectrum oral antibiotic (Cefdinir) for ear infections in the week preceding sample collection. Infants in the analytic sample did not differ significantly from infants in the full sample in age, gender, or feeding type. Recruitment and data collection protocols were approved by the Emory University Institutional Review Board. Secondary analysis was approved by the University of North Carolina at Chapel Hill Institutional Review Board.

**Table 1:** **Characteristics of participating infants at the time of stool sample collection**.

**Infant^a^**	**Sex**	**Age (days)**	**Age (months)**	**Delivery type**	**Daycare**	**Any BM^b^**	**Any Formula^c^**	**EBF^d^**	**Any Solids^e^**	**Age at Solid Introduction (months)**	**Antibiotic Use^f^**	**Birthweight (kg)**	**BMI (kg/m^2^)**
2	F	25	1	C-section	No	Yes	No	Yes	No	5	No	3.09	14.0
		73	2		Yes						No		14.8
		118	4								No		14.8
		156	5								No		16.1
		177	6					No	Yes		No		15.7
		210	7								No		15.1
		289	10								No		14.7
		396	13								No		15.2
3	F	13	0	Vaginal	No	Yes	Yes	No	No	4	No	2.72	9.7
		53	2								No		12.7
		84	3								No		13.7
		181	6			No			Yes		No		14.1
		244	8								No		14.2
4	M	110	4	C-section	No	Yes	Yes	No	No	4	No	4.20	11.4
		124	4								No		12.2
		155	5								No		13.0
		167	5						Yes		No		12.8
		189	6								No		13.0
		252	8								No		13.6
		300	10								No		13.2
		323	11								No		–
7	F	35	1	Vaginal	No	Yes	No	Yes	No	6	No	3.96	15.6
		85	3								No		17.2
		127	4								No		18.3
		150	5								No		–
		192	6					No	Yes		No		19.2
		221	7								No		19.3
		265	9								No		18.7
		305	10								No		19.2
		357	12								No		19.5
		417	14								No		–
8	M	80	3	Vaginal	No	Yes	No	Yes	No	6	No	3.83	15.8
		110	4								No		15.7
		141	5								No		15.2
		191	6								No		15.3
		196	6					No	Yes		No		–
		220	7								No		15.7
		253	8								No		–
10	F	103	3	Vaginal	No	Yes	No	Yes	No	5	No	3.66	14.5
		217	7					No	Yes		No		16.2
		252	8								No		17.2
		279	9								No		17.1
103	M	213	7	Vaginal	Yes	Yes	Yes	No	Yes	5.5	No	3.69	15.9
		255	8			No					No		15.3
		283	9								Yes		15.6
111	M	340	11	Vaginal	Yes	Yes	Yes	No	Yes	4.5	No	3.01	14.9
		402	13								No		14.9
		415	14				No				Yes		–
114	M	323	11	Vaginal	Yes	No	Yes	No	Yes	4.5	No	3.77	15.7
		351	12				No				No		17.3

a Number indicates sample id in original study.

b Indicates whether infant received any breastmilk during the week prior to sample collection.

c Indicates whether infant received formula during the week prior to sample collection.

d Indicates whether infant was exclusively breastfed (receiving only breastmilk and small quantities of medications) during the week prior to sample collection.

e Indicates whether infant received solid foods during the week prior to sample collection.

f Indicates whether infant received antibiotics in the week prior to sample collection. No mothers reported antibiotic use during the study period.

#### Stool sample collection

Parents were asked to retain soiled diapers after each diaper change during a 24-h period. The diapers were placed in individual plastic storage bags labeled for date and time of collection. Bagged diapers were stored in portable coolers equipped with ice packs frozen at −80°C. Diapers were transported to the Laboratory of Reproductive Ecology at Emory University for storage at −80°C within 24-h of collection. Retained portions of the original samples were analyzed in the current study.

#### Infant feeding

Infant feeding type was classified on a weekly-basis using 24-h diet histories where parents were asked to record all the foods and liquids, including specific types and amounts, consumed in the 24-h preceding sample collection. For the present analysis, infants were classified as exclusively breastfed (EBF) if they only received breastmilk and small amounts of medicine or vitamins. Since no infants received formula exclusively, infants were classified as non-exclusively breastfed (non-EBF) if they received both breastmilk and formula. After the introduction of solid foods, which occurred between the 4th and the 6th month of age, infants were categorized into two groups based on whether or not they received infant formula in addition to solids: (1) breastmilk with solids (EBF+S) and (2) formula with solids with or without breastmilk (non-EBF+S).

#### DNA isolation

Isolation of total DNA was performed using a customized protocol in the Qiagen BioRobot Universal (Qiagen) with the Qiagen Blood and Tissue and QIAmp DNA Stool protocols. The Qiagen protocol was modified to ensure isolation of DNA from Gram positive as well as Gram negative bacteria as follows: approximately 100 mg of feces were resuspended in 1.4 ml of ASL buffer, homogeneized in a Tissuelyser (Qiagen) for 2 min at 25 Hz, and heated for 5 min at 95C. Samples were then vortexed (15 s) and centrifuged, and the supernatants were transferred to a new tube. An InhibitEX tablet (Qiagen) was added to remove inhibitors, the mixtures were incubated for 1 min at room temperature, and centrifuged (20,000 × g, 3 min). Two hundred μl of the supernatant were then transferred to a new Eppendorf tube containing 15 μl of proteinase K (600 mAU/ml) and 200 μl of buffer AL were added to the mixture, which was incubated at 70C for 10 min. Two hundred μl of ethanol were then added and the mix was transferred to the bed of the BioRobot Universal and DNA purification was carried out using a customized isolation protocol. DNA was visualized by electrophoresis and quantified using Quant-iT™ PicoGreen^®^ dsDNA Reagent (Molecular Probes, Life Technologies division).

#### 16S rDNA bacterial amplicon pyrosequencing

Initial amplification of the V1-V2 region of the bacterial 16S gene was performed on 50 individual samples from the study as previously described (Devine et al., 2013). Briefly, master mixes for amplicon generation contained the Qiagen Hotstar Hi-Fidelity Polymerase Kit (Qiagen, Valencia CA) with a forward primer composed of the Roche Titanium Fusion Primer A (5′-CCATCTCATCCCTGCGTGTCTCCGACTCAG -3′), a 10 bp Multiplex Identifier (MID) sequence (Roche, Indianapolis, IN) unique to each of the samples and the universal bacteria primer 8F (5′-AGAGTTTGATCCTGGCTCAG-3′) (Edwards et al., 1989). The reverse primer was composed of the Roche Titanium Primer B (5′-CCTATCCCCTGTGTGCCTTGGCAGTCTCAG -3′), the identical 10 bp MID sequence as the forward primer and the reverse bacteria primer 338R (5′-GCTGCCTCCCGTAGGAGT-3′) (Fierer et al., 2008). In order to ensure detection of *Bifidobacterium* species normally highly represented in breastfed babies we used a combination (4:1) of the primers 8F and *Bifidobacterium*-specific Bifido-8F (5′-AGGGTTCGATTCTGGCTCAG-3′) (Martinez et al., 2010)with 335R as the reverse primer as described (Davis et al., 2011). The thermal profile for the amplification of each sample was an initial denaturing step at 94°C for 5 min, followed by a cycling of denaturation at 94°C for 45 s, annealing at 50°C for 30 s, a 1 min 30 s extension at 72°C (35 cycles), a 10 min extension at 72°C and a final hold at 4°C. Each sample was gel-purified individually using the E-Gel Electrophoresis System (Invitrogen, Life Technologies division), quantified using Quant-iT™ PicoGreen^®^ dsDNA Reagent, and the concentration was standardized. The 16S rDNA amplicons from the pooled sample were sequenced on a Roche GS FLX Titanium instrument (Microbiome Core Facility, Chapel Hill NC). Initial data analysis, base pair calling and trimming to yield high quality reads, were performed by Research Computing at UNC.

#### Amplicon high-throughput sequencing data analysis

This bioproject has been registered at the National Center for Biotechnology Information (NCBI) under BioProject ID PRJNA270898. Analysis of sequencing data was carried out using the QIIME pipeline (Caporaso et al., 2010) as described (Devine et al., 2013). The combined raw sequencing data plus metadata describing the samples were de-multiplexed and filtered for quality control. Next, data was denoised using the recommended denoising method (Denoiser, Caporaso et al., 2010). Sequences were grouped into OTUs (Operational Taxonomic Units) at a 97% level using Uclust (Edgar, 2010). After taxonomic assignation of OTUs, sequences were aligned and phylogenetic trees were built (Price et al., 2010). QIIME was also used to calculate alpha diversity on rarefied OTU tables to assess sampling depth coverage using observed species, Shannon, and phylogenetic diversity (PD) metrics. These metrics were used to quantify microbiome community structure with species richness (S) providing a measure of the number of different bacterial species present per sample and phylogenetic diversity (PD) providing a measure of the diversity of taxa present. To correct for different numbers of sequences in each sample, we randomly subsampled 2908 sequences in each sample (the number of sequences in the sample with the lowest number of sequences). To evaluate the similarities between bacterial communities, a combination of Unifrac significance and principal coordinate analysis (PCoA) using Fast Unifrac (Lozupone et al., 2006) were performed to compare samples based on relevant parameters (Table 2). Supervised machine-learning technique Random Forests (RF) within QIIME (Knights et al., 2011) were used to identify bacterial genera and species-level OTUs that differentiate the community composition between feeding groups.

**Table 2:** **Analysis of similarities (ANOSIM) between parameters and categories evaluated in this study**.

**Parameter**	**Categories**	***P*-value**	***R*-value**
**Age**	**Under 5, over 5 months**	**0.003**	**0.1608**
Feeding1	Any breast milk (Yes-No)	0.180	0.1123
**Feeding2**	**Any formula (Yes-No)**	**0.003**	**0.1579**
**Feeding3**	**Any solids (Yes-No)**	**0.002**	**0.1693**
Feeding4	Exclusively breastfed (EBF) plus solids (Yes-No)	0.868	−0.0613
**Feeding5**	**Non-exclusively breastfed (non-EBF) plus solids (Yes-No)**	**0.009**	**0.1784**
**Feeding Group**	**EBF, non-EBF, EBF+S, non-EBF+S**	**0.001**	**0.2394**
**Feeding by Age1**	**Exclusively breastfed younger than 5.5 months (Yes-No)**	**0.023**	**0.1846**
Feeding by Age2	Non-exclusively breastfed younger than 5.5 months (Yes-No)	0.193	0.1041
**Daycare**	**Yes-No**	**0.008**	**0.1870**
**Sex**	**Female, Male**	**0.011**	**0.0791**

Alpha was set at 0.05.

Bold values indicate factors that had a weak (ANOSIM R < 0.2, P < 0.05) or medium (ANOSIM R = 0.2 to 0.5, P < 0.05) statistically significant impact on variables.

#### PICRUSt analysis of 16S amplicon sequencing data

PICRUSt is a tool designed to infer metagenomic information from 16S amplicon sequencing data (Langille et al., 2013). In the present study, analysis of 16S amplicon sequencing data was performed using the default settings of PICRUSt (version 0.9.1). The resulting metagenomic data were entered into the HMP unified metabolic analysis network (HUMAnN) (Abubucker et al., 2012) pipeline (version 0.98) to sort individual genes into Kyoto encyclopedia of genes and genomes (KEGG) pathways representing varying proportions of each imputed sample metagenome.

### Experimental design

Forty-nine stool samples from 9 infants were divided in four groups to investigate differences between EBF and non-EBF infants before and after the introduction of solid foods: (a) exclusively breastfed infants before introduction of solid foods (EBF, *n* = 12, infant age 25–191 days), (b) non-exclusively breastfed infants before introduction of solid foods (non-EBF, *n* = 6, infant age 13–155 days), (c) EBF infants after introduction of solid foods (EBF+S, *n* = 17, infant age 177–396 days), and (d) non-EBF infants after introduction of solid foods (non-EBF+S, *n* = 14, infant age 167–415 days). Secondarily, we tested whether, within these groups, infants differed by daycare attendance (in home vs. out of home care).

#### Statistical analyses

We used the non-parametric test ANOSIM (Analysis of Similarities) within QIIME to test whether two or more groups of samples were significantly different (Fierer et al., 2010). The number of permutations was set at 999 to calculate *p*-values. To identify bacterial taxa and biological pathways predicted by PICRUSt that were significantly (*p* ≤ 0.05) over or under represented in the different groups we applied the Steel-Dwass All Pairs test in JMP genomics (SAS JMP Genomics 5.0).

## Results

Amplicon pyrosequencing of stool samples yielded a total of 304,175 reads with an average of 6083 reads per sample. Sequences were assigned to 814 Operational Taxonomic Units (OTUs) at ≥97% similarity clustering into 110 genera, 20 classes, and 9 phyla. Bacterial relative abundance at phylum level, phylogenetic diversity (PD), bacterial gene functional representation, feeding type and collection time point for individual infants are shown in Supplemental Figure 2.

### Bacterial diversity was influenced by age, feeding, and daycare

Analysis of similarities (ANOSIM) showed statistically significant (*p* < 0.05) differences in the infant gut microbiome due to age, feeding, and daycare attendance (Table 2), indicating that the compositional dissimilarities between the groups were higher than those within the groups. Regardless of the feeding type, analysis of the stool microbiome composition of samples collected over time reflected an increment of the average bacterial Phylogenetic Diversity (PD) and species richness (S) during the first year. Infants aged 12–14 months had the highest number of species (*S* = 71.4 ± 29.5, Supplemental Figure 1A) and diversity values (*PD* = 8.76 ± 2.5, Supplemental Figure 1B).

**Figure 1:**
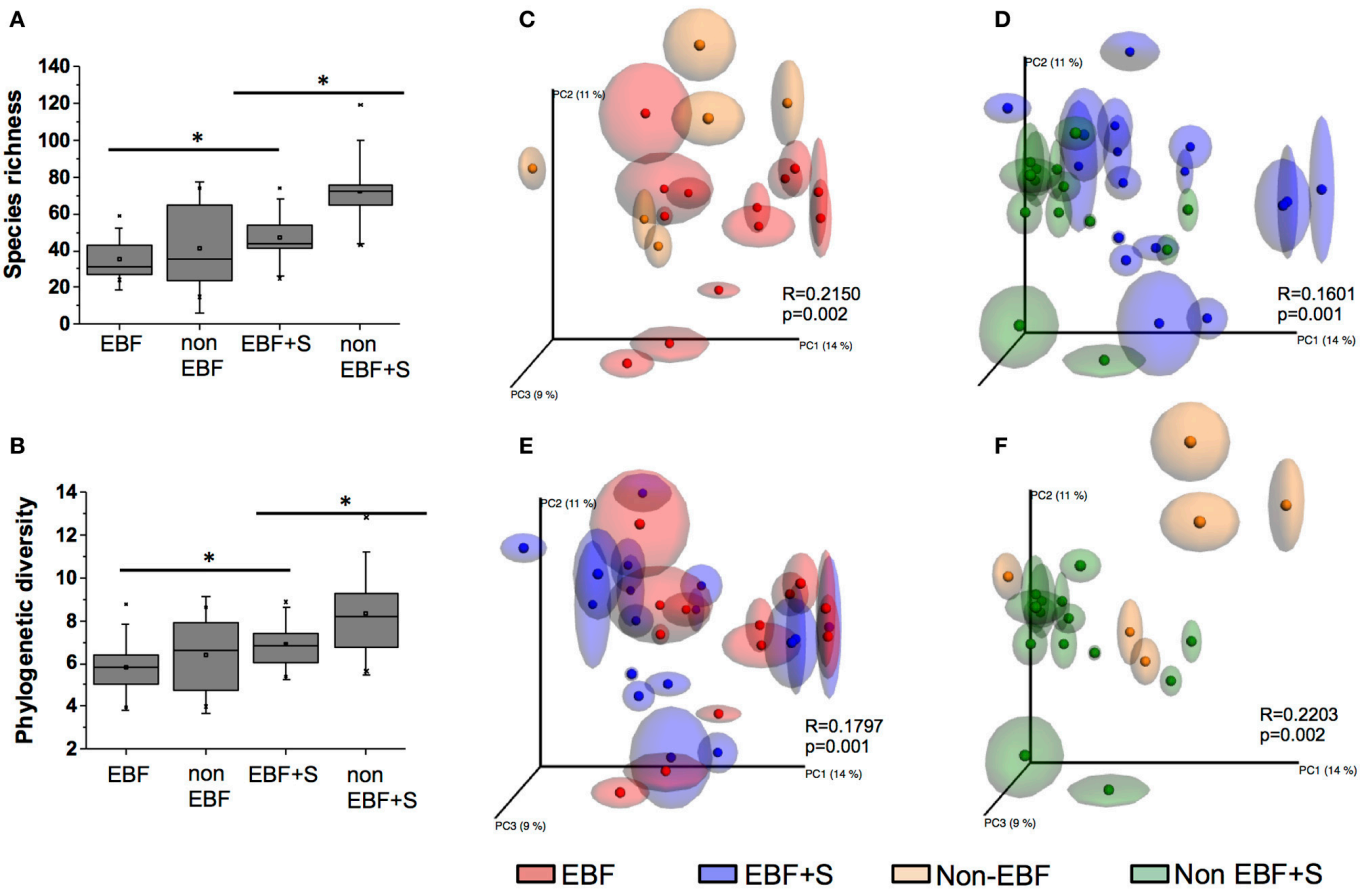
**(A)** A comparison of species richness S and **(B)** phylogenetic diversity PD between feeding groups. Non-EBF infants showed significantly higher S and PD values than EBF infants, and both values were impacted by the introduction of solid foods (^*^p < 0.05). **(C–F)** Comparison of unweighted UniFrac PCoA plots with repeated resampling (jackknifing) of microbiota from different feeding groups. ANOSIM R and P values are indicated in each figure.

We observed significant differences in species diversity and richness between the groups (*p* < 0.05 for all comparisons). EBF infants had the lowest species richness and diversity (*PD* = 5.8 ± 1.3, *S* = 35.3 ± 11.2). Species richness and diversity was higher in non-EBF infants (*PD* = 6.4 ± 1.8, *S* = 41.5 ± 23.8) and EBF+S infants (*PD* = 6.9 ± 1.1, *S* = 47.11 ± 14.9) reaching the highest values in non-EBF+S infants (*PD* = 8.3 ± 2.0, *S* = 71.9 ± 18.7) (Figures 1A,B).

Principal coordinates analysis (PCoA) of unweighted UniFrac (Lozupone and Knight, 2005) also identified significant differences in the infant stool microbiome according to feeding pattern. Samples from EBF infants segregated from samples from non-EBF infants (Figure 1C). This segregation continued after solid foods were introduced into the diet (Figure 1D). Although EBF samples significantly clustered away from EBF+S samples (Figure 1E), these clusters were less defined (*R* = 0.18) than those observed between non-EBF vs. non-EBF+S samples (*R* = 0.22; Figure 1F).

**Figure 2:**
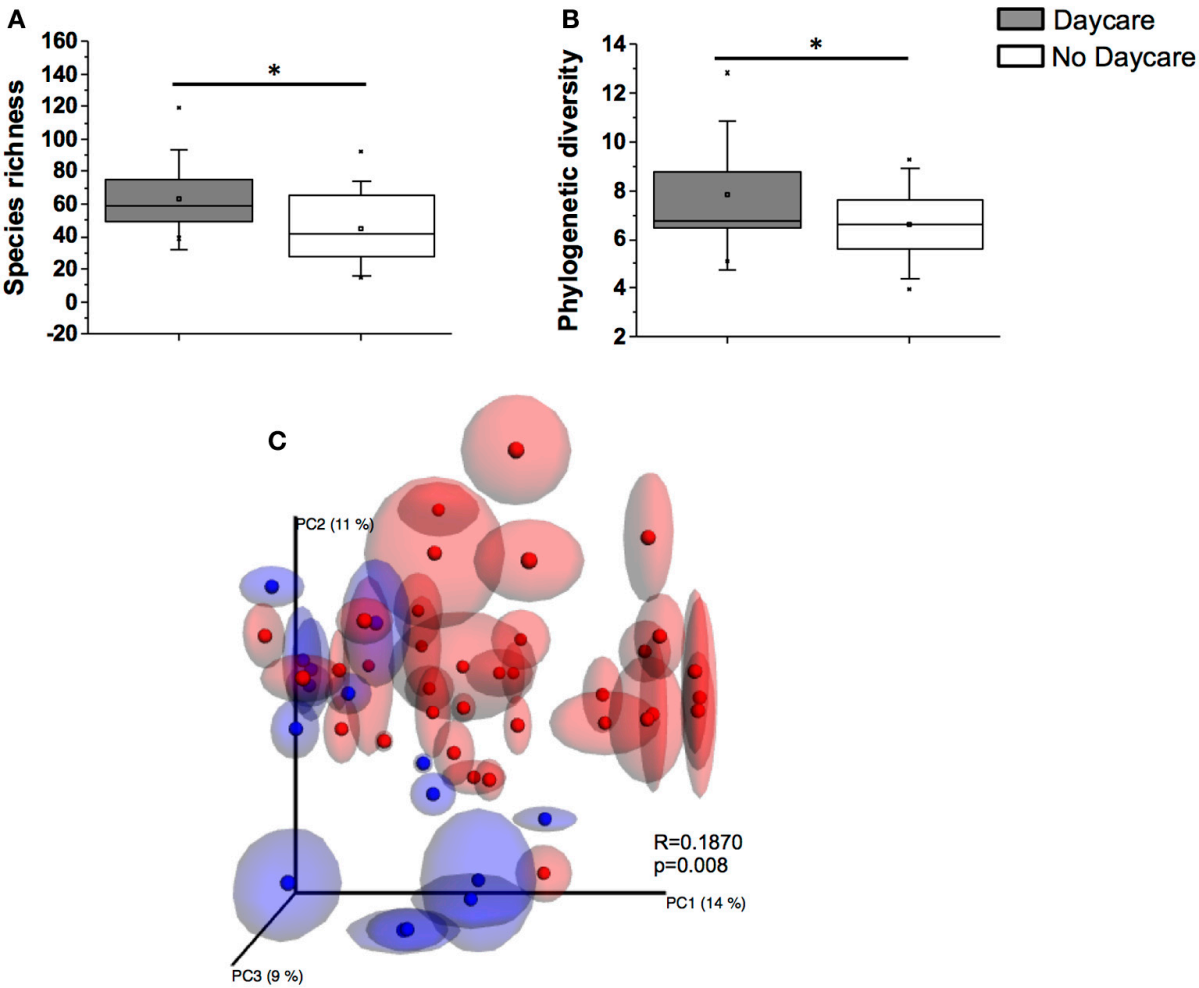
A comparison of species richness S **(A)** and phylogenetic diversity PD **(B)** between infants attending daycare and infants cared for at home (^*^p < 0.05). (C) PCoA analysis of samples with repeated resampling (jackknifing) according to daycare attendance regardless of feeding group. Blue symbols represent samples from infants attending daycare. Red symbols represent infants not attending daycare. ANOSIM R and P values are indicated in the figure.

Infants in out-of-home daycare had significantly higher diversity and species richness values (*PD* = 7.7 ± 2.0, *S* = 63.1 ± 20.4) compared to infants cared for in home (*PD* = 6.6 ± 1.5, *S* = 44.8 ± 19.2, *p* < 0.05), regardless of the feeding type (Figures 2A,B). A significant clustering pattern was also observed in PCoA plots by daycare attendance (Figure 2C).

### Differences in the composition of the stool microbiome of EBF and non-EBF infants

**Figure 3:**
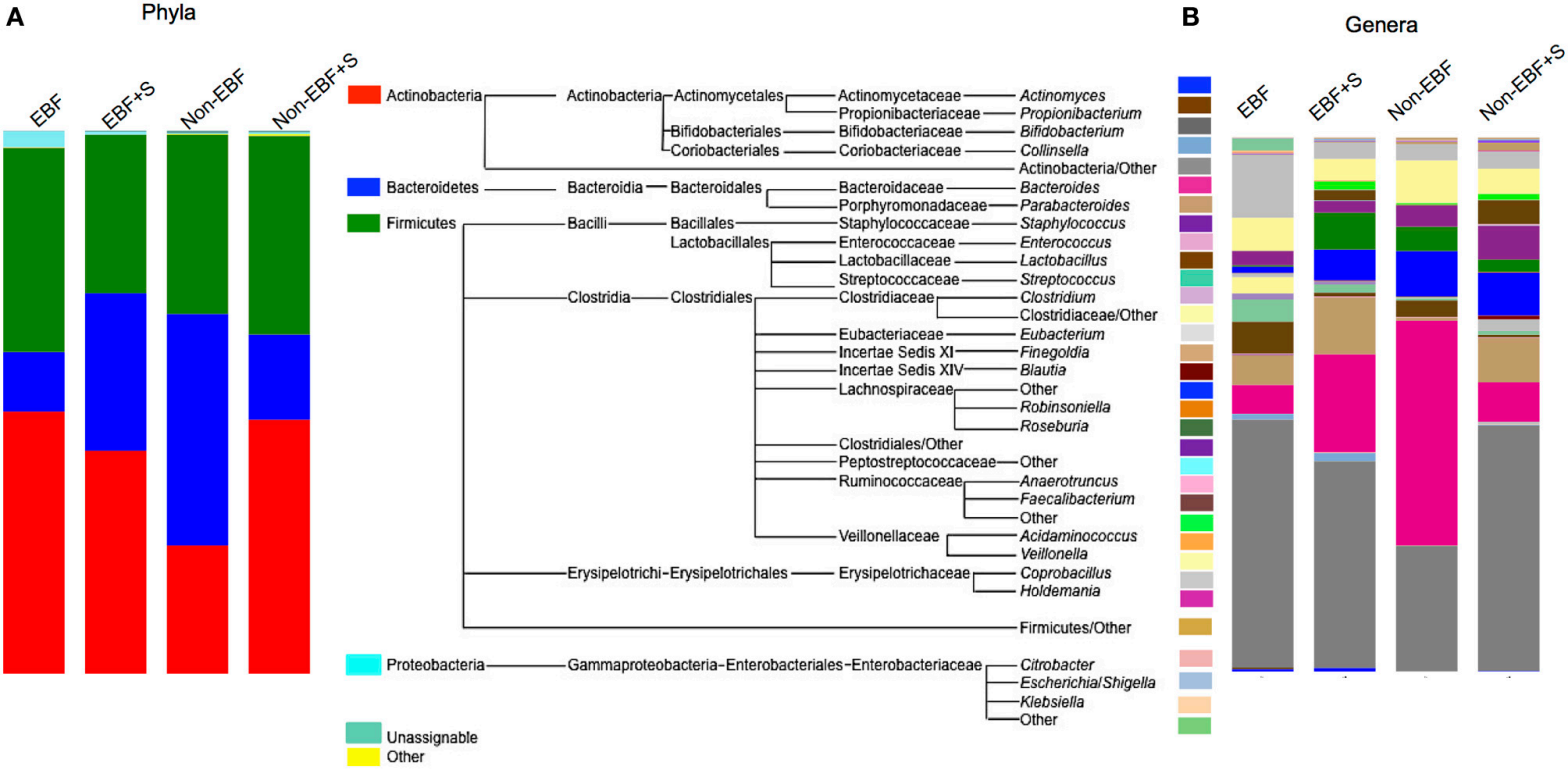
**Relative abundance of (A) phyla and (B) genera in the different feeding groups**. The mid panel shows a diagram of the taxa represented in the samples. For clarity only genera represented over 0.1% were included. The total representation per sample was >99.9%.

The stool microbiome of EBF and non-EBF infants was characterized by different phyla proportions. Actinobacteria were significantly over represented in EBF infants (average relative abundance 48.3%, *p* = 0.055) while Bacteroidetes were over represented in non-EBF infants (42.6%, *p* = 0.054). No significant differences were detected between groups in the Firmicutes phylum (37.6% in EBF, 33% in non-EBF infants) (Figure 3A, Table 3) Within the phyla, several taxa differed significantly between feeding groups. Within the phylum Actinobacteria, the relative abundance of *Bifidobacterium* was lower in non-EBF infants than in EBF infants, while *Eggerthella* was more abundant in the non-EBF group. *Bacteroides* within the phylum Bacteroidetes was significantly more abundant in non-EBF infants, though these differences did not reach statistical significance. Within the phylum Firmicutes, abundance of Clostridiales, Lachnospiraceae, *Blautia* and *Faecalibacterium* was significantly higher (*p* < 0.05) in non-EBF compared to EBF infants (Table 3).

**Table 3:** **Significant differences in relative abundances (%) between feeding groups**.

	**EBF compared to non-EBF**			**EBF+S compared to non-EBF+S**	
	**EBF**	**non-EBF**		**EBF-S**	**non-EBF+S**
Actinobacteria^*^	48.3 ± 34.3	23.6 ± 28.2	**Firmicutes**	29.2 ± 23.6	36.6 ± 19.6
Eggerthella	1.4e − 3 ± 6.0e − 3	0.04 ± 0.05	Blautia^*^	0.08 ± 0.2	0.6 ± 1.0
Bifidobacterium	46.4 ± 35.2	23.5 ± 28.2	Anaerotruncus^*^	0.0 ± 0.0	0.2 ± 0.2
Bacteroidetes^*^	11.0 ± 22.5	42.6 ± 42.7	Eubacterium^*^	0.03 ± 0.07	2.1
Bacteroides^*^	5.4 ± 14.6	42.1 ± 43.3	Ruminococcaceae^*^	1.6 ± 4.8	1.1 ± 1.3
Firmicutes	37.6 ± 31.2	33.0 ± 25.9	Clostridiales^*^	2.2 ± 8.0	6.3 ± 5.6
Clostridiales^*^	2.7 ± 7.6	4.0 ± 8.1	Faecalibacterium^*^	1.9 ± 6.4	4.4
Lachnospiraceae^*^	1.2 ± 3.3	8.5 ± 14.1	Lactobacillus^*^	0.7 ± 3.1	0.5 ± 2.7
Blautia^*^	0.0 ± 0.0	0.06 ± 0.16			
Faecalibacterium^*^	1.4e − 4 ± 6.2e − 3	0.16 ± 0.26			
	**EBF compared to EBF+S**			**non-EBF compared to non-EBF+S**	
	**EBF**	**EBF+S**		**non-EBF**	**non-EBF+S**
Actinobacteria	48.3 ± 34.3	41.1 ± 34.2	**Actinobacteria§**	23.6 ± 28.2	46.8 ± 26.5
Eggerthella^*^	1.4e − 3 ± 6.0e − 3	0.07 ± 0.16	*Bifidobacterium§*	23.5 ± 28.2	46.0 ± 26.7
Bacteroidetes§	11.0 ± 22.5	29.0 ± 30.5	**Firmicutes**	33.0 ± 25.9	36.6 ± 19.6
Bacteroides§	5.4 ± 14.6	18.3 ± 27.1	Ruminococcaceae^*^	0.3 ± 1.0	1.1 ± 1.2
Firmicutes	37.6 ± 31.2	29.2 ± 23.6	Blautia^*^	0.06 ± 0.16	0.6 ± 1.0
Peptostreptococcaceae^*^	1.4e − 3 ± 6.8e − 3	0.1 ± 0.2	*Oscillibacter§*	0.0 ± 0.0	0.08 ± 0.2
Blautia^*^	0.0 ± 0.0	0.08 ± 0.2	**Proteobacteria**	0.5 ± 0.74	0.3 ± 0.4
Proteobacteria	2.93 ± 5.4	0.5 ± 0.74	*Escherichia/Shiggella*§	0.0 ± 0.0	0.2 ± 0.4
Staphylococcus^*^	0.18 ± 0.7	0.0 ± 0.0			
Roseateles^*^	0.02 ± 0.04	0.0 ± 0.0			
Neisseria^*^	8.1e − 3 ± 0.02	0.0 ± 0.0			
Escherichia/Shigella^*^	6.7e − 3 ± 0.02	0.3 ± 0.7			

Asterisks indicate p-values were ≤ 0.05; section signs indicate p ≤ 0.1. Phyla are in bold, with lower taxonomic levels indicated below.

Random Forests (RF) analysis revealed distinct community signatures for EBF and non-EBF infants (baseline error = 0.24, cross-validation error = 0.21 ± 0.09). We considered an OTU to be highly predictive if its importance score was at least 0.001, and in our study 60 OTUs were identified as highly predictive (Supplemental Table 1). Of the 60 highly predictive OTUs, 17 corresponded to the phylum Actinobacteria and 12 specifically to the genus *Bifidobacterium*. Other highly predictive genera included *Streptococcus* (5 OTUs), *Veillonella* (3 OTUs), *Parabacteroides* (3 OTUs), *Eggerthella, Bacteroides, Actinomyces, Enterococcus*, and *Coprobacillus* (each with 2 OTUs).

### Gut microbiome compositional impact of the introduction of solid foods

The introduction of solid foods into the diet of EBF infants resulted in a marked, significant increase in the abundance of Bacteroidetes, especially *Bacteroides* with a concomitant, though non-statistically significant, reduction in the relative abundance of Firmicutes and Actinobacteria (Figure 3, Table 3). At the genus level we observed significantly decreased abundance of *Staphylococcus* and *Roseateles* and increased abundance of *Eggerthella*, *Blautia*, and Peptostreptococcaceae after incorporation of solid foods in EBF infants (Table 3). The introduction of solid foods into the diet of non-EBF infants was associated with an increased abundance of Actinobacteria (*p* = 0.06), specifically *Bifidobacterium* (Figure 3, Table 3). Within the phylum Firmicutes, Ruminococcaeceae and *Blautia* increased significantly after the introduction of solids.

RF analysis identified distinct community signatures for EBF+S and non-EBF+S infants (baseline error = 0.45, cross-validation error = 0.12 ± 0.15). In both groups Actinobacteria was the dominant phylum, followed by Firmicutes and Bacteroides (Figure 3). However, analysis at the genus level showed that abundance of *Lactobacillus* and Ruminococcaceae was significantly higher in EBF+S infants. Non-EBF+S infants showed significantly higher levels of Clostridiales, *Blautia, Faecalibacterium, Anaerotruncus*, and *Eubacterium* (Figure 3, Table 3).

### A cohort core microbiome defined by breastfeeding

The core microbiome, defined as “organisms common across microbiomes hypothesized to play a key role in ecosystem function within a habitat” (Turnbaugh et al., 2007), contains members common to two or more microbial assemblages associated with a condition such as feeding pattern. In this study, the core microbiome in EBF infants included *Bifidobacterium* and *Coprobacillus*, which were present in 75% of the samples. In contrast, *Veillonella* and Clostridiales were present in 100% and *Bacteroides* in 75% of non-EBF infants (Figure 5). Incorporation of solid foods into the diet of EBF infants was translated into the presence, in 75% of samples, of *Veillonella, Roseburia* and the family Lachnospiraceae in addition to the persistent species identified in EBF samples. Introduction of solid foods to the diet of non-EBF infants resulted in the identification of *Bifidobacterium, Streptococcus*, and *Coprobacillus* in the microbiome of 75% of non-EBF infants.

**Figure 4:**
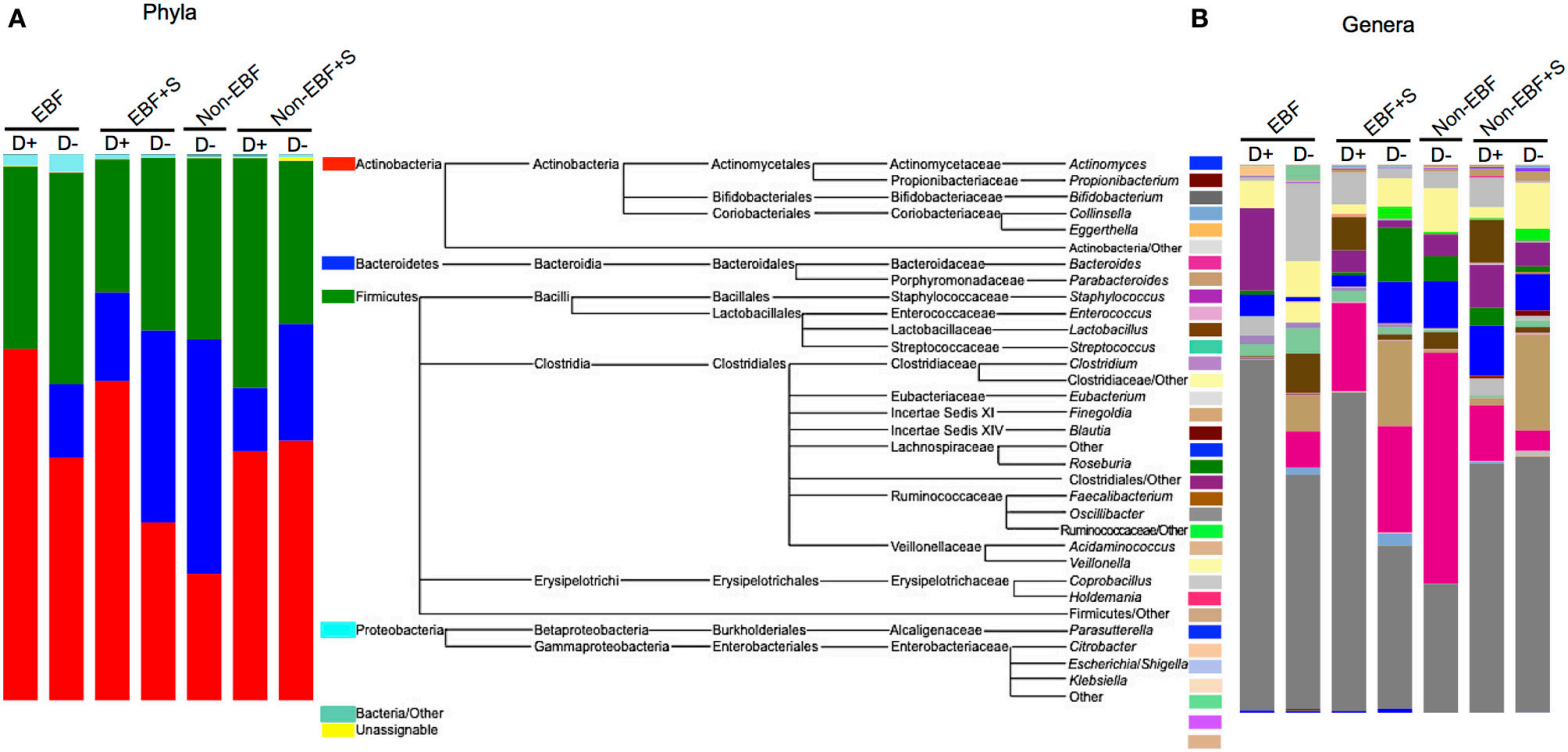
**Relative abundance of (A) phyla and (B) genera by feeding group and attendance to daycare (D+)**. The mid panel shows a diagram of the taxa represented in the samples. For clarity only genera represented over 0.1% were included. The total representation per sample was >99.9%.

**Figure 5:**
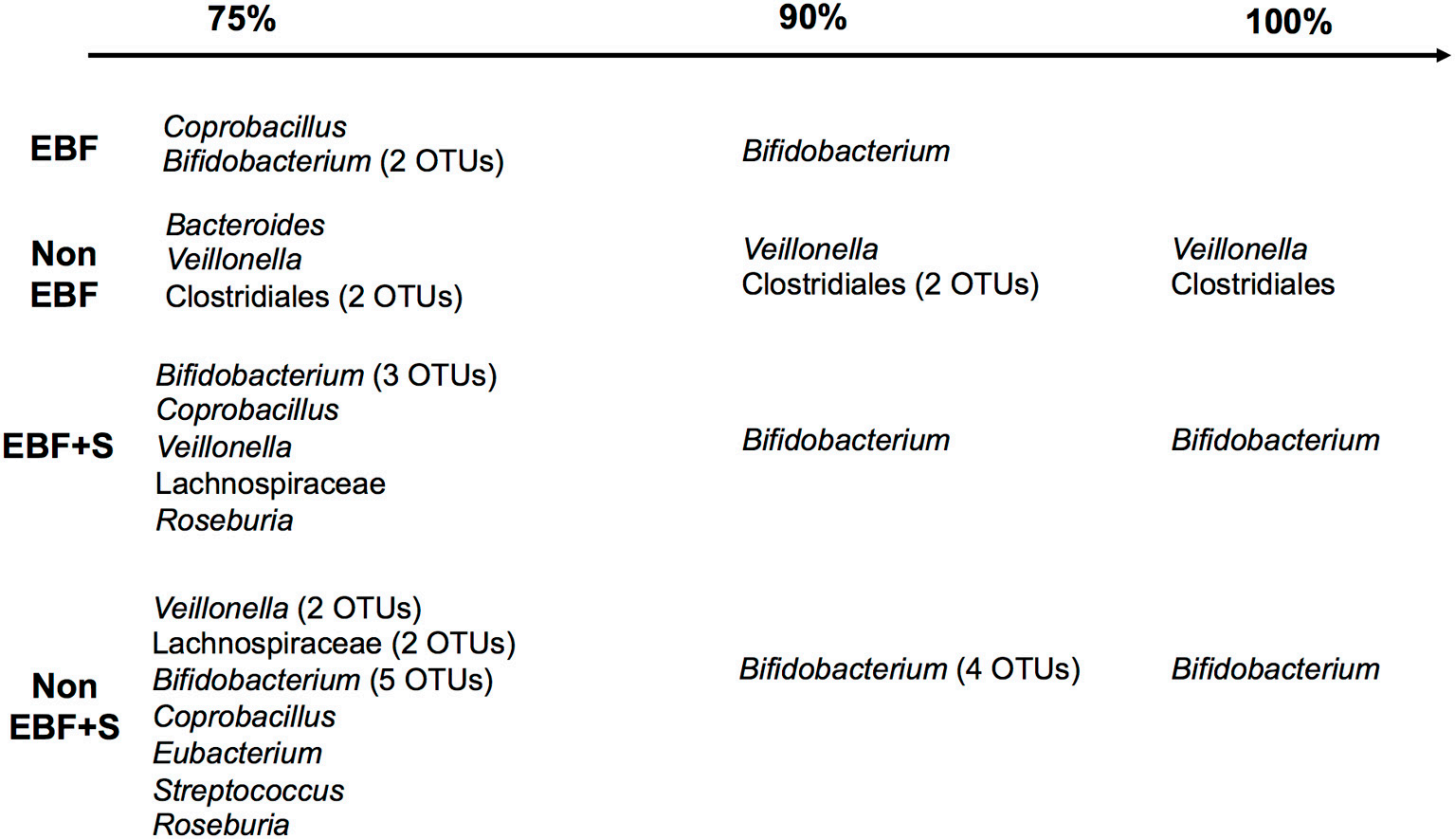
**The infant core microbiome by feeding group**. The percent of samples in which each taxa was identified is shown on the top.

### Gut microbiome composition and daycare attendance

The secondary aim of our study was to investigate the impact of daycare attendance on the community structure of the infant gut microbiome. Due to the low number of infants in our cohort we were not able to stratify each group by age. Nonetheless, we observed specific differences in EBF infants attending daycare compared to infants that were cared for at home, with significantly increased Firmicutes in the first group, specifically *Eubacterium*, Clostridiales, Ruminococcaceae, *Lactobacillus*, *Roseburia*, and Lachnospiraceae, and decreased *Bacteroides*. EBF+S infants who attended daycare had also a significant increased representation of Firmicutes, specifically *Eubacterium*, *Acidaminococcus*, and species of the family Clostridiaceae, as well as the Proteobacteria *Sutterella*. (Figure 4, Supplemental Figure 3). No non-EBF infants attended daycare; consequently we were not able to compare daycare attendance in relation to this feeding group, but we did observe an increased abundance of the taxa *Faecalibacterium*, *Anaerotruncus*, *Coprobacillus*, and Clostridiales in the phylum Firmicutes, *Collinsella* in the phylum Actinobacteria, and Enterobacteriaceae in the phylum Proteobacteria, and decreased abundance of genera of the family Ruminococcaceae in the non-EBF+S attending daycare compared to infants in the same group that did not attend daycare.

### Metabolic functions associated with feeding and feeding transitions

**Figure 6:**
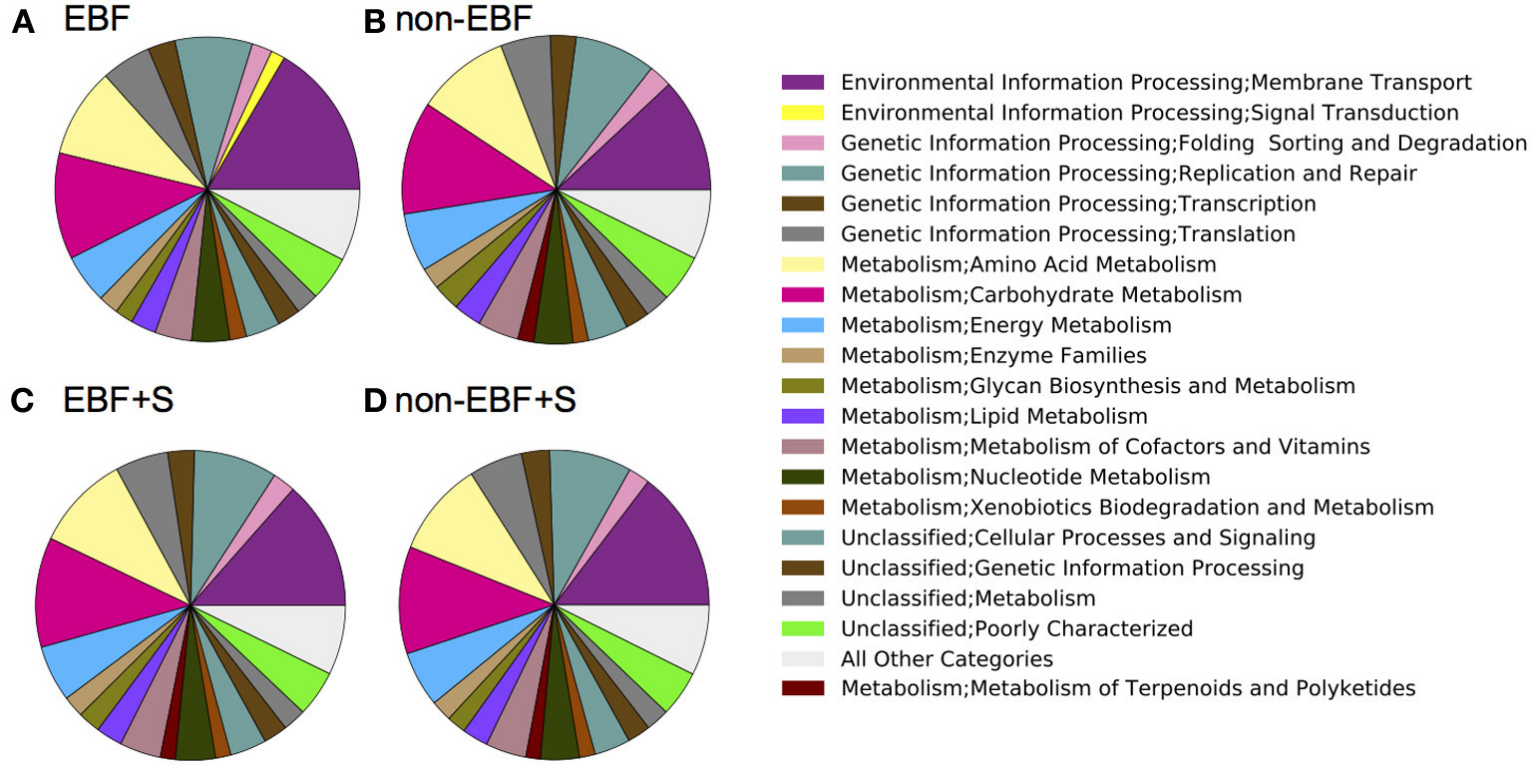
**PICRUSt (Langille et al., 2013) predicted summary of COG categories from 16S amplicon pyrosequencing**. Results are depicted by feeding group (**A**, Exclusively breastfed; **B**, Non-exclusively breastfed; **C**, Exclusively breastfed plus solids; and **D**, Non-exclusively breastfed plus solids) and show an over representation of pathways involved in environmental information processing, specifically membrane transport, and an under representation of pathways involved in the metabolism of terpenoids and polyketides in EBF infants.

PICRUSt analysis identified 328 different functional pathways, and showed that, despite the inter- and intra-individual variation in the composition of the fecal microbiota, samples were predicted to have similar functional profiles (Supplemental Figure 2). Overall, our analysis revealed differences in the mean relative predicted abundance of gene categories (clusters of orthologous groups, COGs) and metabolic pathways (KEGG) between feeding groups. Reflecting their lower diversity, EBF infants had the lowest number of KEGG metabolic pathways representative genes, followed by non-EBF, non-EBF+S and EBF+S infants. Samples from EBF infants, however, had an overrepresentation of pathways involved in environmental information processing, specifically membrane transport (Figure 6). EBF samples showed a significantly increased (*p* = 0.01) abundance of genes encoding sugar-specific phosphotransferase system (PTS) components like fructose, N acetilglucosamine, trehalose and glucose transporters. In addition the PTS system ascorbate-specific IIB component was also enriched in EBF infants.

**Figure 7:**
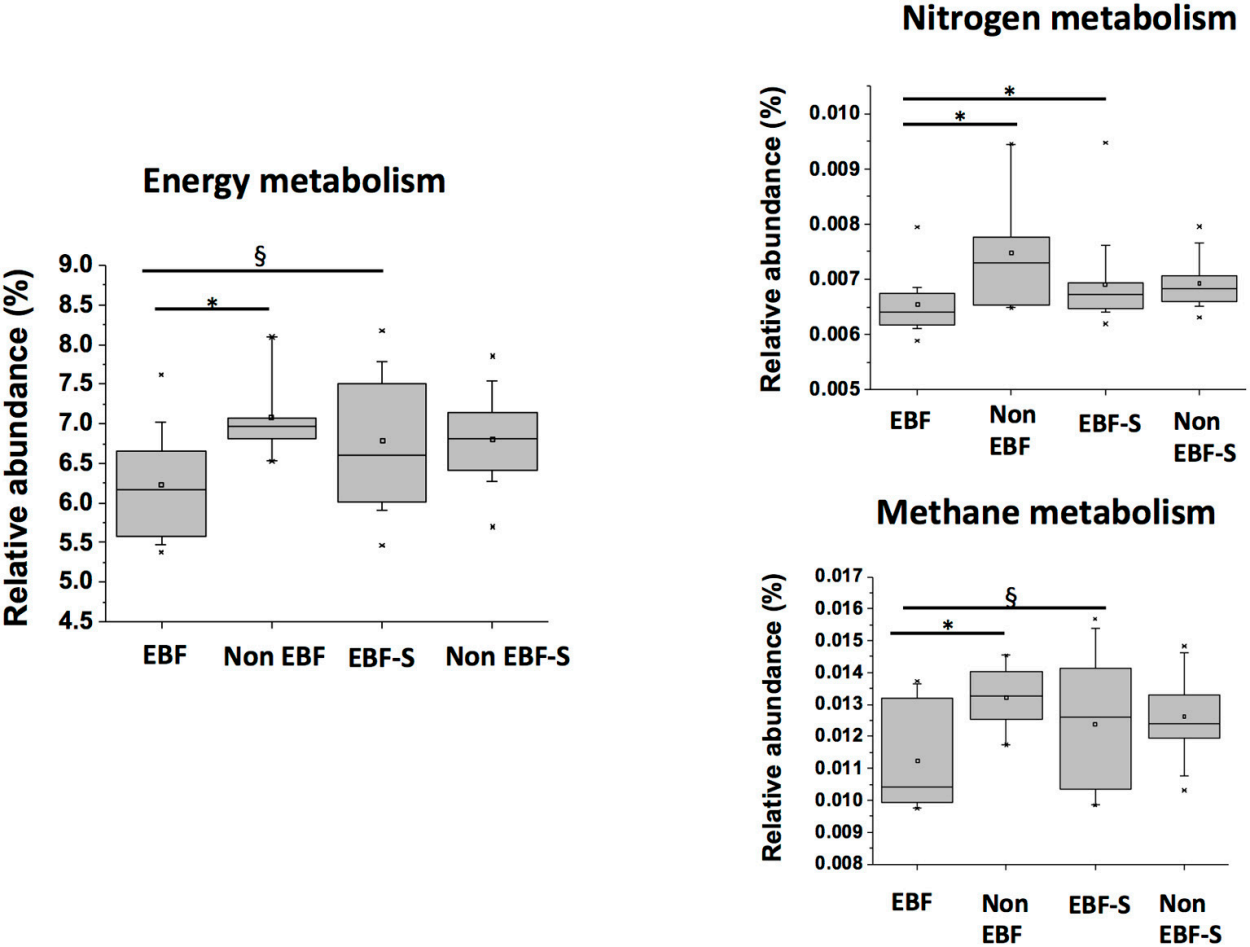
Relative abundances of KEGG pathways involved in energy metabolism, and nitrogen and methane within energy metabolism in the different feeding groups (^*^p < 0.05, §*p* < 0.1).

In contrast, energy metabolism, specifically nitrogen and methane metabolism genes, were overrepresented in non-EBF and EBF+S samples compared to EBF infants (*p* ≤ 0.05), probably due to the higher protein content of formula and solid foods (Figure 7). Stool samples from non-EBF infants also had a significantly higher predicted abundance (*p* ≤ 0.05) of genes involved in protein catabolism like D-aminopeptidases, tripeptide aminopeptidases, aminoacylhistidine dipeptidases, dipeptidases D, and g-D-glutamyl-meso-diaminopimelate peptidases.

When we compared EBF to EBF+S samples to assess the impact of introduction of solid foods, we observed a significantly increased predicted representation of the Metabolism category, specifically biosynthesis of other secondary metabolites, energy metabolism and metabolism of terpenoids and polyketides, in EBF+S samples. Enzymatic functions significantly overrepresented in EBF+S infants included quinate/shikimate dehydrogenase (EC:1.1.1.282), involved in phenylalanine, tyrosine and tryptophan biosynthesis, oxalyl-CoA decarboxylase (EC:4.1.1.8), pyruvate ferredoxin oxidoreductase (alpha and gamma subunits) involved in several essential metabolic pathways including glycolysis, polyphosphate kinase (EC:2.7.4.1) involved in oxidative phosphorylation and RNA degradation, starch phosphorylase (EC:2.4.1.1) involved in starch and sucrose metabolism and insulin signaling pathway, and pyruvate ferredoxin oxidoreductase (K00169 and K00172, alpha and gamma subunits), responsible for the oxidative decarboxylation of pyruvate to acetyl-coenzyme A in anaerobic bacteria (Chabriere et al., 1999).

A comparison of non-EBF and non-EBF+S samples showed an increased predicted representation of the pathways involved in cellular processes (cell growth and death), genetic information processing (replication and repair), human diseases (immune system diseases), and organismal systems (endocrine system) after the introduction of solid foods. Two hundred and thirty genes were overrepresented in non-EBF+S infants compared to non-EBF infants compared to only 24 in the EBF+S to EBF comparison group. Enzymatic functions overrepresented in the non-EBF+S included several methyl-accepting chemotaxis proteins (MCP) including serine sensor, aspartate sensor receptor, ribose and galactose sensor, and peptide sensor receptor, involved in signal transduction (Williams and Stewart, 1999), and the curli assembly protein including all components: major curlin subunit (K04334, csgA), minor curlin subunit (K04335, csgB), curli production protein (K04336, csgC), curli production assembly/transport component CsgE (K04337, csgE), curli production assembly/transport component CsgF (K04338, csgF), and curli production assembly/transport component CsgG (K06214, csgG). Curli are extracellular fibers expressed by species of *Escherichia* and *Salmonella* involved in colonization and biofilm formation (Chapman et al., 2002; Vidal et al., 1998). Also overrepresented in the non-EBF+S group compared to the non-EBF group were genes involved in the biosynthesis of colanic acid (K03207, colanic acid biosynthesis protein WcaH and K03208, colanic acid biosynthesis protein WcaI), an exopolysaccharide common to many enterobacteria (Stevenson et al., 1996) and aerobactin synthesis genes (K03894, aerobactin synthetase subunit alpha and K03895, aerobactin synthetase subunit beta), a bacterial virulence factor that allows *Escherichia coli* to sequester iron in iron-depleted environments (de Lorenzo et al., 1986).

## Discussion

The infant gut microbiota develops from birth until maturity, establishing a mutually beneficial cohabitation with the host. As a consequence, the infant gut microbiome is less stable than the adult microbiome and susceptible to modulation or disruption by environmental assault. Our study aimed to identify differences in the intestinal microbiome between exclusively breastfed and non-exclusively breastfed infants in a relatively small cohort of 9 healthy infants, who were followed from 2 weeks to 14 months of age before and after the introduction of solid foods, and between infants cared for in out-of-home daycare centers and those cared for at home. We found a lower diversity in the microbiota of younger infants, regardless of feeding type, which increased over time, confirming previous small and large cohorts studies that demonstrated that diversity increases from 0 to 3 years of age (Koenig et al., 2011; Yatsunenko et al., 2012). Attendance in out-of-home daycare was identified as a discriminant parameter in the composition of the gut by the ANOSIM analysis. Infants in out-of-home care showed greater diversity in the microbiota compared to those cared for in the home, regardless of feeding type. To the best of our knowledge, our study is the first to report the microbiome analysis and identification of increased species richness and diversity in infants attending daycare.

Along with these differences by age and daycare attendance, we also found significant differences across feeding patterns. Non-EBF infants displayed a higher diversity and species richness than EBF infants. Clustering analysis indicated that EBF and non-EBF groups had different overall microbiome composition. Our results confirm earlier studies (Mountzouris et al., 2002; Palmer et al., 2007; Koenig et al., 2011; Gomez-Llorente et al., 2013) showing that the gut microbiome of EBF infants was different than the microbiome of non-EBF infants, with increased proportions of *Bifidobacterium* and lower abundance of Bacteroidetes and Clostridiales. Importantly, we found that introduction of solid foods into to diet was associated with more marked changes in the microbiota composition of non-EBF infants, differences that were also reflected in the mean abundances of predicted gene categories and metabolic pathways.

In our study, non-EBF+S samples showed the highest PD values, followed by EBF+S, non-EBF and EBF samples. PCoA plots of unweighted UniFrac analysis showed that samples from EBF infants tended to segregate from samples from non-EBF infants and this tendency continued after solid foods were introduced into the diet. Interestingly, although EBF samples tended to separate from EBF+S samples, less defined clusters suggested fewer differences in the stool microbiota between these groups when solid foods were incorporated into the diet. On the contrary, we observed very defined clusters when we compared non-EBF vs. non-EBF+S samples suggesting a higher impact of solid foods on the microbiome of non-EBF babies. Many studies refer to the microbiome of formula-fed infants as “adult-like” (Harmsen et al., 2000; Olivares et al., 2013); however, our data suggest that the microbiome of those infants is different to the microbiome of EBF infants as well as the adult microbiome. Further, the introduction of solid foods appeared to have a more dramatic impact in non-EBF than in EBF infants, where breast milk has prepared the host and the gut microbiome for the introduction of simple vegetal food (Matamoros et al., 2013). We hypothesize that the non-EBF microbiome could be less adaptable when faced with new food substrates, since formula composition does not vary over time while breast milk composition varies according to the mother's diet and other factors (Coppa et al., 1993; Cabrera-Rubio et al., 2012). The apparent paradox of a low diversity, high plasticity gut microbiome certainly warrants further investigation.

We conducted Random Forests (RF) analysis to identify specific bacterial taxa highly predictive of feeding-type and feeding transition. RF has been recently used in microbiome analysis because RF classification models are versatile, have a high prediction accuracy and provide additional information such as variable importances (Touw et al., 2013). RF identified 60 highly predictive bacterial taxa with 28% from the the Actinobacteria, 12 specifically from the genus *Bifidobacterium*. The dominant taxa in EBF infants were Actinobacteria followed by Firmicutes whereas the microbiome of non-EBF infants was dominated by Bacteroidetes and Firmicutes. Specifically, EBF infants had a higher relative abundance of *Bifidobacterium* species compared to non-EBF infants where *Bacteroides* species were dominant. Dominance of *Bifidobacterium* in EBF vs. non-EBF infants has been debated since numerous studies have found a lower abundance of *Bifidobacterium* in formula-fed infants relative to breast-fed infants (Yoshioka et al., 1983; Favier et al., 2002; Hopkins et al., 2005), but other studies have reported *Bifidobacterium* as the dominant group in both groups (Yatsunenko et al., 2012; Gomez-Llorente et al., 2013). Considering the high interindividual variation we might hypothesize that both groups are Colonized early by *Bifidobacterium*, but the abundance of these microorganisms is not favored by formula, while it is maintained in exclusively breastfed babies by the presence of oligosaccharides in human milk.

Discernible predicted metabolic differences were observed between EBF and non-EBF infants, with increased representation of sugar transporters in EBF samples compared to non-EBF samples, which are essential for the digestion of milk carbohydrates (Candela et al., 2012). Conversely, non-EBF samples were enriched in genes involved in energy metabolism, specifically nitrogen and methane metabolism genes as well as peptidases, probably due to the higher protein content of formula. The PTS system ascorbate-specific IIB component was also enriched in EBF infants. This is in accordance with different studies that showed a higher content of vitamin C in breast fed infants than formula fed infants (Tlaskal and Novakova, 1990; Aycicek et al., 2006). Kurokawa et al. (2007) observed in an early study that the infant microbiome was dominated by COGs included in the Carbohydrate transport and metabolism, including 12 families of glycosylhydrolases. They also identified several enzymes involved in degradation of non-digestible polysaccharides of plant origin.

Human milk is composed of lactose (70 g/L), followed by lipids (40 g/L). Interestingly, the third most abundant component are human milk oligosaccharides (HMO; 5–15 g/L), followed by protein at 8 g/L (Zivkovic et al., 2011). When infants switch from breastfeeding to solids, the fraction of energy from protein increases rapidly (Zivkovic et al., 2011). Accordingly, when we compared EBF to EBF+S samples to assess the impact of introduction of solid foods, we observed a significantly increased representation of the Metabolism category, specifically biosynthesis of other secondary metabolites, energy metabolism and metabolism of terpenoids and polyketides in EBF+S samples. Yet, as reflected in our Unifrac PCoA analysis that suggested subdued changes in the microbiome of EBF infants when solids were introduced into their diet, only 24 enzymes were over represented in EBF+S infants compared to the EBF group. Of interest are the overrepresentation of the biotin operon repressor BirA (K03524), which regulates transcription of the prokaryotic biotin operon (Wilson et al., 1992), the thiamine biosynthesis lipoprotein (K03734) involved in the conversion of aminoimidazole ribotide (AIR), a purine intermediate, to the 4-amino-5-hydroxymethyl-2-methyl pyrimidine (HMP) moiety of thiamine in *Salmonella* (Beck and Downs, 1998), and the oxalyl-CoA decarboxylase involved in oxalate degradation (Azcarate-Peril et al., 2006). Overrepresentation of these pathways reflects the adaptation of the microbiome to availability of microbionutrients due to introduction of new foods.

In contrast, over 200 bacterial gene categories were overrepresented in non-EBF+S compared to non-EBF infants including several methyl-accepting chemotaxis proteins (MCP) including serine sensor, aspartate sensor receptor, ribose and galactose sensor, and peptide sensor receptor, involved in signal transduction (Williams and Stewart, 1999). Signal-responsive components of transmembrane signal-transducing regulatory systems include methyl-accepting chemotaxis proteins and membrane-bound, two-component histidine kinases. Prokaryotes use these regulatory networks to channel environmental cues into adaptive responses. Also overrepresented were all curlin components, extracellular fibers expressed by species of *Escherichia* and *Salmonella* involved in colonization and biofilm formation (Vidal et al., 1998; Chapman et al., 2002). Such pathway differences may underlie the long standing findings that breastfeeding is associated with lower morbidity from a number of diseases including acute otitis media, atopic dermatitis, gastrointestinal infections, lower respiratory tract diseases, asthma, obesity, cardiovascular diseases, type 1 and type 2 diabetes, childhood leukemia, and Sudden Infant Death Syndrome (SIDS) in infants in developed countries (Ip et al., 2007).

Despite the limitations of PICRUSt as a predictor of gene functionality from 16S amplicon sequencing data, we found similarities between our results and previously reported metagenomic sequencing (Koenig et al., 2011; Vaishampayan et al., 2011; Schwartz et al., 2012). Two previous studies reported the over representation of genes involved in sugar transport of human milk glycans like phosphotransferase system for N-acetiylglucosamine and glucose and for other carbohydrates like fructose in EBF infants (Koenig et al., 2011; Vaishampayan et al., 2011). Koenig et al. (2011) also reported the enrichment of genes involved in vitamin and cofactor biosynthesis after the introduction of solids in one EBF infant. This is in accordance with our results in which we detected the overrepresentation of genes involved in biosynthesis of biotine and thiamine when solids were introduced into the diet of EBF infants. Finally, Schwartz et al. (2012), found virulence characteristics that differed between breast-fed and formula fed infants. Among others, they detected an enrichment of genes involved in iron scavenging. This is in agreement with our report of an over-representation of genes involved in the synthesis of aerobactin, an iron-chelating agent, in non-EBF+solids infants.

Our collection of infant stool samples as part of prospective, longitudinal study of “normal” infant growth and development expands previous descriptive research (Koenig et al., 2011). Further, the high rates of breastfeeding permitted examination of the effects of mixed breast- and formula-feeding, a group that has been less often described in the literature, and the impact of solid food introduction in EBF and non-EBF infants. Our use of 24-h food diaries at each sample collection likely reduced misclassification of infant feeding type based on recall. Along with these strengths, the present study has a number of limitations. Due to the small sample available for secondary analysis, we were not able to test the impact of other characteristics known to shape the infant microbiome, such as birth type (vaginal vs. Caesarian-section) or antibiotic use. While this sample size is relatively small, our fine-grained analysis permits hypothesis generation for larger studies to assess the bacterial and functional impacts of solid food introduction and daycare attendance. Additionally, we used PICRUSt to estimate metagenomic information rather than directly measuring the metagenome. Consequently, our findings of metabolic differences between the groups reflect predicted, rather than actual, function; nevertheless, PICRUSt results have been validated against metagenomic sequencing and show correlations of 80–90% in human studies (Langille et al., 2013).

Our study showed that feeding and feeding-transitions have significant, dramatic impacts on the infant gut microbiome and that out-of-home day care attendance may further influence microbial diversity independently of feeding. These results indicate that the early nutrient and pathogenic environment may shape colonization during this highly plastic and sensitive period. Our findings that different genetic and metabolic pathways were activated in EBF and non-EBF infants, even among infants that were still predominantly receiving breastmilk, further suggest that feeding-based differences in microbiome composition have the potential to contribute to the programming of infant metabolism and immune function. Such programming during the critical period of weaning with the transition from a milk-based to a more varied, solid-based diets characterized by higher levels of carbohydrates and animal proteins may have long-term consequences for not only the establishment of the adult microbiome but also the development of metabolic diseases like obesity, diabetes, cardiovascular disease, and non-alcoholic fatty liver disease (NAFLD).

## Author contributions

All authors contributed to study design and interpretation. Amanda L. Thompson and Michelle L. Lampl acquired the samples. Andrea Monteagudo-Mera and Maria B. Cadenas conducted DNA isolation and sample processing for sequencing. Andrea Monteagudo-Mera and M. A. Azcarate-Peril conducted data analysis. Amanda L. Thompson, Andrea Monteagudo-Mera and M. A. Azcarate-Peril drafted the manuscript. All authors provided critical revision to the manuscript and have approved the final version. All authors agree to take responsibility for accuracy and integrity of the research.

### Conflict of interest statement

The authors declare that the research was conducted in the absence of any commercial or financial relationships that could be construed as a potential conflict of interest.

## Supplementary material

Supplemental Figure 1 **Bacterial Phylogenetic Diversity (PD) and species richness (S) during the first 14 months of age increases over time**.

Supplemental Figure 2 **Relative abundance of bacterial phyla phylogenetic diversity (PD), bacterial gene functional representation, feeding type and collection time points in stool samples by individual infant**.

Supplemental Figure 3 **Relative abundances of bacterial taxa over- and under-represented in infants attending daycare by feeding group**.

Supplemental Table 1 **List of predictive OTUs generated by Random forests–supervised classification analysis**.

## References

[B1] Abubucker S. Segata N. Goll J. Schubert A. M. Izard J. Cantarel B. L.. (2012). Metabolic reconstruction for metagenomic data and its application to the human microbiome. PLoS Comput. Biol. 8:e1002358. 10.1371/journal.pcbi.1002358 22719234 PMC3374609

[B2] Amarri S. Benatti F. Callegari M. L. Shahkhalili Y. Chauffard F. Rochat F.. (2006). Changes of gut microbiota and immune markers during the complementary feeding period in healthy breast-fed infants. J. Pediatr. Gastroenterol. Nutr. 42, 488–495. 10.1097/01.mpg.0000221907.14523.6d 16707969

[B3] Augustine J. M. Crosnoe R. L. Gordon R. (2013). Early child care and illness among preschoolers. J. Health Soc. Behav. 54, 315–334. 10.1177/0022146513496106 23956356 PMC4556116

[B4] Aycicek A. Erel O. Kocyigit A. Selek S. Demirkol M. R. (2006). Breast milk provides better antioxidant power than does formula. Nutrition 22, 616–619. 10.1016/j.nut.2005.12.011 16635560

[B5] Azcarate-Peril M. A. Bruno-Barcena J. M. Hassan H. M. Klaenhammer T. R. (2006). Transcriptional and functional analysis of oxalyl-coenzyme A (CoA) decarboxylase and formyl-CoA transferase genes from Lactobacillus acidophilus. Appl. Environ. Microbiol. 72, 1891–1899. 10.1128/AEM.72.3.1891-1899.2006 16517636 PMC1393175

[B6] Beck B. J. Downs D. M. (1998). The apbE gene encodes a lipoprotein involved in thiamine synthesis in Salmonella typhimurium. J. Bacteriol. 180, 885–891. 9473043 10.1128/jb.180.4.885-891.1998 PMC106968

[B7] Cabrera-Rubio R. Collado M. C. Laitinen K. Salminen S. Isolauri E. Mira A. (2012). The human milk microbiome changes over lactation and is shaped by maternal weight and mode of delivery. Am. J. Clin. Nutr. 96, 544–551. 10.3945/ajcn.112.037382 22836031

[B8] Candela M. Biagi E. Maccaferri S. Turroni S. Brigidi P. (2012). Intestinal microbiota is a plastic factor responding to environmental changes. Trends Microbiol. 20, 385–391. 10.1016/j.tim.2012.05.003 22672911

[B9] Caporaso J. G. Kuczynski J. Stombaugh J. Bittinger K. Bushman F. D. Costello E. K.. (2010). QIIME allows analysis of high-throughput community sequencing data. Nat. Methods 7, 335–336. 10.1038/nmeth.f.303 20383131 PMC3156573

[B10] Chabriere E. Charon M. H. Volbeda A. Pieulle L. Hatchikian E. C. Fontecilla-Camps J. C. (1999). Crystal structures of the key anaerobic enzyme pyruvate:ferredoxin oxidoreductase, free and in complex with pyruvate. Nat. Struct. Biol. 6, 182–190. 10.1038/5870 10048931

[B11] Chapman M. R. Robinson L. S. Pinkner J. S. Roth R. Heuser J. Hammar M.. (2002). Role of Escherichia coli curli operons in directing amyloid fiber formation. Science 295, 851–855. 10.1126/science.1067484 11823641 PMC2838482

[B12] Coppa G. V. Gabrielli O. Pierani P. Catassi C. Carlucci A. Giorgi P. L. (1993). Changes in carbohydrate composition in human milk over 4 months of lactation. Pediatrics 91, 637–641. 8441573

[B13] Davis L. M. Martinez I. Walter J. Goin C. Hutkins R. W. (2011). Barcoded pyrosequencing reveals that consumption of galactooligosaccharides results in a highly specific bifidogenic response in humans. PLoS ONE 6:e25200. 10.1371/journal.pone.0025200 21966454 PMC3180383

[B14] De Filippo C. Cavalieri D. Di Paola M. Ramazzotti M. Poullet J. B. Massart S.. (2010). Impact of diet in shaping gut microbiota revealed by a comparative study in children from Europe and rural Africa. Proc. Natl. Acad. Sci. U.S.A. 107, 14691–14696. 10.1073/pnas.1005963107 20679230 PMC2930426

[B15] de Lorenzo V. Bindereif A. Paw B. H. Neilands J. B. (1986). Aerobactin biosynthesis and transport genes of plasmid ColV-K30 in Escherichia coli K-12. J. Bacteriol. 165, 570–578. 2935523 10.1128/jb.165.2.570-578.1986 PMC214457

[B16] Devine A. A. Gonzalez A. Speck K. E. Knight R. Helmrath M. A. Lund P. K.. (2013). Impact of ileocecal resection and concomitant antibiotics on the microbiome of the murine jejunum and colon. PLoS ONE 8:e73140. 10.1371/journal.pone.0073140 24015295 PMC3754918

[B17] Donnet-Hughes A. Perez P. F. Dore J. Leclerc M. Levenez F. Benyacoub J.. (2010). Potential role of the intestinal microbiota of the mother in neonatal immune education. Proc. Nutr. Soc. 69, 407–415. 10.1017/S0029665110001898 20633308

[B18] Edgar R. C. (2010). Search and clustering orders of magnitude faster than BLAST. Bioinformatics 26, 2460–2461. 10.1093/bioinformatics/btq461 20709691

[B19] Edwards U. Rogall T. Blocker H. Emde M. Bottger E. C. (1989). Isolation and direct complete nucleotide determination of entire genes. Characterization of a gene coding for 16S ribosomal RNA. Nucleic Acids Res. 17, 7843–7853. 10.1093/nar/17.19.7843 2798131 PMC334891

[B20] Fallani M. Amarri S. Uusijarvi A. Adam R. Khanna S. Aguilera M.. (2011). Determinants of the human infant intestinal microbiota after the introduction of first complementary foods in infant samples from five European centres. Microbiology 157(Pt 5), 1385–1392. 10.1099/mic.0.042143-0 21330436

[B21] Fanaro S. Chierici R. Guerrini P. Vigi V. (2003). Intestinal microflora in early infancy: composition and development. Acta Paediatr. 91, 48–55. 10.1111/j.1651-2227.2003.tb00646.x 14599042

[B22] Favier C. F. Vaughan E. E. De Vos W. M. Akkermans A. D. (2002). Molecular monitoring of succession of bacterial communities in human neonates. Appl. Environ. Microbiol. 68, 219–226. 10.1128/AEM.68.1.219-226.2002 11772630 PMC126580

[B23] Fierer N. Hamady M. Lauber C. L. Knight R. (2008). The influence of sex, handedness, and washing on the diversity of hand surface bacteria. Proc. Natl. Acad. Sci. U.S.A. 105, 17994–17999. 10.1073/pnas.0807920105 19004758 PMC2584711

[B24] Fierer N. Lauber C. L. Zhou N. McDonald D. Costello E. K. Knight R. (2010). Forensic identification using skin bacterial communities. Proc. Natl. Acad. Sci. U.S.A. 107, 6477–6481. 10.1073/pnas.1000162107 20231444 PMC2852011

[B25] Gill S. R. Pop M. Deboy R. T. Eckburg P. B. Turnbaugh P. J. Samuel B. S.. (2006). Metagenomic analysis of the human distal gut microbiome. Science 312, 1355–1359. 10.1126/science.1124234 16741115 PMC3027896

[B26] Gomez-Llorente C. Plaza-Diaz J. Aguilera M. Munoz-Quezada S. Bermudez-Brito M. Peso-Echarri P.. (2013). Three main factors define changes in fecal microbiota associated with feeding modality in infants. J. Pediatr. Gastroenterol. Nutr. 57, 461–466. 10.1097/MPG.0b013e31829d519a 23752082

[B27] Greiner T. Backhed F. (2011). Effects of the gut microbiota on obesity and glucose homeostasis. Trends Endocrinol. Metab. 22, 117–123. 10.1016/j.tem.2011.01.002 21353592

[B28] Guarner F. Malagelada J. R. (2003). Gut flora in health and disease. Lancet 361, 512–519. 10.1016/S0140-6736(03)12489-0 12583961

[B29] Gutierrez-Castrellon P. Lopez-Velazquez G. Diaz-Garcia L. Jimenez-Gutierrez C. Mancilla-Ramirez J. Estevez-Jimenez J.. (2014). Diarrhea in preschool children and lactobacillus reuteri: a randomized controlled trial. Pediatrics 133, e904–e909. 10.1542/peds.2013-0652 24639271

[B30] Harmsen H. J. Wildeboer-Veloo A. C. Raangs G. C. Wagendorp A. A. Klijn N. Bindels J. G.. (2000). Analysis of intestinal flora development in breast-fed and formula-fed infants by using molecular identification and detection methods. J. Pediatr. Gastroenterol. Nutr. 30, 61–67. 10.1097/00005176-200001000-00019 10630441

[B31] Hopkins M. J. Macfarlane G. T. Furrie E. Fite A. Macfarlane S. (2005). Characterisation of intestinal bacteria in infant stools using real-time PCR and northern hybridisation analyses. FEMS Microbiol. Ecol. 54, 77–85. 10.1016/j.femsec.2005.03.001 16329974

[B32] Ip S. Chung M. Raman G. Chew P. Magula N. DeVine D. (2007). Breastfeeding and Maternal and Infant Health Outcomes in Developed Countries. Evidence Report/Technology Assessment No. 153. Rockville, MD: Agency for Healthcare Research and Quality. (Prepared by Tufts-New England Medical Center Evidence-based Practice Center, under Contract No. 290-02-0022). AHRQ Publication No. 07-E007. PMC4781366 17764214

[B33] Jeurink P. V. van Bergenhenegouwen J. Jimenez E. Knippels L. M. Fernandez L. Garssen J.. (2013). Human milk: a source of more life than we imagine. Benef. Microbes 4, 17–30. 10.3920/BM2012.0040 23271066

[B34] Knights D. Costello E. K. Knight R. (2011). Supervised classification of human microbiota. FEMS Microbiol. Rev. 35, 343–359. 10.1111/j.1574-6976.2010.00251.x 21039646

[B35] Koenig J. E. Spor A. Scalfone N. Fricker A. D. Stombaugh J. Knight R.. (2011). Succession of microbial consortia in the developing infant gut microbiome. Proc. Natl. Acad. Sci. U.S.A. 108(Suppl. 1), 4578–4585. 10.1073/pnas.1000081107 20668239 PMC3063592

[B36] Kurokawa K. Itoh T. Kuwahara T. Oshima K. Toh H. Toyoda A.. (2007). Comparative metagenomics revealed commonly enriched gene sets in human gut microbiomes. DNA Res. 14, 169–181. 10.1093/dnares/dsm018 17916580 PMC2533590

[B37] Langille M. G. Zaneveld J. Caporaso J. G. McDonald D. Knights D. Reyes J. A.. (2013). Predictive functional profiling of microbial communities using 16S rRNA marker gene sequences. Nat. Biotechnol. 31, 814–821. 10.1038/nbt.2676 23975157 PMC3819121

[B38] Lozupone C. Hamady M. Knight R. (2006). UniFrac—an online tool for comparing microbial community diversity in a phylogenetic context. BMC Bioinformatics 7:371. 10.1186/1471-2105-7-371 16893466 PMC1564154

[B39] Lozupone C. Knight R. (2005). UniFrac: a new phylogenetic method for comparing microbial communities. Appl. Environ. Microbiol. 71, 8228–8235. 10.1128/AEM.71.12.8228-8235.2005 16332807 PMC1317376

[B40] Marques T. M. Wall R. Ross R. P. Fitzgerald G. F. Ryan C. A. Stanton C. (2010). Programming infant gut microbiota: influence of dietary and environmental factors. Curr. Opin. Biotechnol. 21, 149–156. 10.1016/j.copbio.2010.03.020 20434324

[B41] Martinez I. Kim J. Duffy P. R. Schlegel V. L. Walter J. (2010). Resistant starches types 2 and 4 have differential effects on the composition of the fecal microbiota in human subjects. PLoS ONE 5:e15046. 10.1371/journal.pone.0015046 21151493 PMC2993935

[B42] Matamoros S. Gras-Leguen C. Le Vacon F. Potel G. de La Cochetiere M. F. (2013). Development of intestinal microbiota in infants and its impact on health. Trends Microbiol. 21, 167–173. 10.1016/j.tim.2012.12.001 23332725

[B43] Mountzouris K. C. McCartney A. L. Gibson G. R. (2002). Intestinal microflora of human infants and current trends for its nutritional modulation. Br. J. Nutr. 87, 405–420. 10.1079/BJN2002563 12010580

[B44] Nauta A. J. Ben Amor K. Knol J. Garssen J. van der Beek E. M. (2013). Relevance of pre- and postnatal nutrition to development and interplay between the microbiota and metabolic and immune systems. Am. J. Clin. Nutr. 98, 586S–593S. 10.3945/ajcn.112.039644 23824726

[B45] Nielsen S. Nielsen D. S. Lauritzen L. Jakobsen M. Michaelsen K. F. (2007). Impact of diet on the intestinal microbiota in 10-month-old infants. J. Pediatr. Gastroenterol. Nutr. 44, 613–618. 10.1097/MPG.0b013e3180406a11 17460496

[B46] Olivares M. Laparra J. M. Sanz Y. (2013). Host genotype, intestinal microbiota and inflammatory disorders. Br. J. Nutr. 109(Suppl. 2), S76–S80. 10.1017/S0007114512005521 23360883

[B47] Orrhage K. Nord C. E. (1999). Factors controlling the bacterial colonization of the intestine in breastfed infants. Acta Paediatr. 88, 47–57. 10569223 10.1111/j.1651-2227.1999.tb01300.x

[B48] Palmer C. Bik E. M. DiGiulio D. B. Relman D. A. Brown P. O. (2007). Development of the human infant intestinal microbiota. PLoS Biol. 5:e177. 10.1371/journal.pbio.0050177 17594176 PMC1896187

[B49] Parrett A. M. Edwards C. A. (1997). *In vitro* fermentation of carbohydrate by breast fed and formula fed infants. Arch. Dis. Child. 76, 249–253. 10.1136/adc.76.3.249 9135267 PMC1717116

[B50] Price M. N. Dehal P. S. Arkin A. P. (2010). FastTree 2—approximately maximum-likelihood trees for large alignments. PLoS ONE 5:e9490. 10.1371/journal.pone.0009490 20224823 PMC2835736

[B51] Schwartz S. Friedberg I. Ivanov I. V. Davidson L. A. Goldsby J. S. Dahl D. B.. (2012). A metagenomic study of diet-dependent interaction between gut microbiota and host in infants reveals differences in immune response. Genome Biol. 13:r32. 10.1186/gb-2012-13-4-r32 22546241 PMC3446306

[B52] Stevenson G. Andrianopoulos K. Hobbs M. Reeves P. R. (1996). Organization of the Escherichia coli K-12 gene cluster responsible for production of the extracellular polysaccharide colanic acid. J. Bacteriol. 178, 4885–4893. 8759852 10.1128/jb.178.16.4885-4893.1996 PMC178271

[B53] Tannock G. W. Lawley B. Munro K. Gowri Pathmanathan S. Zhou S. J. Makrides M.. (2013). Comparison of the compositions of the stool microbiotas of infants fed goat milk formula, cow milk-based formula, or breast milk. Appl. Environ. Microbiol. 79, 3040–3048. 10.1128/AEM.03910-12 23455335 PMC3623157

[B54] Thompson A. L. Lampl M. (2013). Prenatal and postnatal energetic conditions and sex steroids levels across the first year of life. Am. J. Hum. Biol. 25, 643–654. 10.1002/ajhb.22424 23904043 PMC4271319

[B55] Tlaskal P. Novakova V. (1990). The effect of natural and artificial nutrition on vitamin C and E levels in the normal infant. Cesk. Pediatr. 45, 402–407. 2289268

[B56] Touw W. G. Bayjanov J. R. Overmars L. Backus L. Boekhorst J. Wels M.. (2013). Data mining in the Life Sciences with Random Forest: a walk in the park or lost in the jungle? Brief. Bioinformatics 14, 315–326. 10.1093/bib/bbs034 22786785 PMC3659301

[B57] Turnbaugh P. J. Ley R. E. Hamady M. Fraser-Liggett C. M. Knight R. Gordon J. I. (2007). The human microbiome project. Nature 449, 804–810. 10.1038/nature06244 17943116 PMC3709439

[B58] Vael C. Desager K. (2009). The importance of the development of the intestinal microbiota in infancy. Curr. Opin. Pediatr. 21, 794–800. 10.1097/MOP.0b013e328332351b 19770768

[B59] Vaishampayan P. A. Kuehl J. V. Froula J. L. Morgan J. L. Ochman H. Francino M. P. (2011). Comparative metagenomics and population dynamics of the gut microbiota in mother and infant. Genome Biol. Evol. 2, 53–66. 10.1093/gbe/evp057 20333224 PMC2839348

[B60] Vidal O. Longin R. Prigent-Combaret C. Dorel C. Hooreman M. Lejeune P. (1998). Isolation of an Escherichia coli K-12 mutant strain able to form biofilms on inert surfaces: involvement of a new ompR allele that increases curli expression. J. Bacteriol. 180, 2442–2449. 9573197 10.1128/jb.180.9.2442-2449.1998 PMC107187

[B61] Williams S. B. Stewart V. (1999). Functional similarities among two-component sensors and methyl-accepting chemotaxis proteins suggest a role for linker region amphipathic helices in transmembrane signal transduction. Mol. Microbiol. 33, 1093–1102. 10.1046/j.1365-2958.1999.01562.x 10510225

[B62] Wilson K. P. Shewchuk L. M. Brennan R. G. Otsuka A. J. Matthews B. W. (1992). Escherichia coli biotin holoenzyme synthetase/bio repressor crystal structure delineates the biotin- and DNA-binding domains. Proc. Natl. Acad. Sci. U.S.A. 89, 9257–9261. 10.1073/pnas.89.19.9257 1409631 PMC50105

[B63] Wu G. D. Chen J. Hoffmann C. Bittinger K. Chen Y. Y. Keilbaugh S. A.. (2011). Linking long-term dietary patterns with gut microbial enterotypes. Science 334, 105–108. 10.1126/science.1208344 21885731 PMC3368382

[B64] Yatsunenko T. Rey F. E. Manary M. J. Trehan I. Dominguez-Bello M. G. Contreras M.. (2012). Human gut microbiome viewed across age and geography. Natre 486, 222–227. 10.1038/nature11053 22699611 PMC3376388

[B65] Yoshioka H. Iseki K. Fujita K. (1983). Development and differences of intestinal flora in the neonatal period in breast-fed and bottle-fed infants. Pediatrics 72, 317–321 6412205

[B66] Zivkovic A. M. German J. B. Lebrilla C. B. Mills D. A. (2011). Human milk glycobiome and its impact on the infant gastrointestinal microbiota. Proc. Natl. Acad. Sci. U.S.A. 108(Suppl. 1), 4653–4658. 10.1073/pnas.1000083107 20679197 PMC3063602

