# Supplementary figures and images for: Milk- and solid-feeding practices and daycare attendance are associated with differences in bacterial diversity, predominant communities, and metabolic and immune function of the infant gut microbiome
Source: Front Cell Infect Microbiol. 2015 Feb 5;5:3. doi: 10.3389/fcimb.2015.00003 (PMC4318912; doi:10.3389/fcimb.2015.00003)

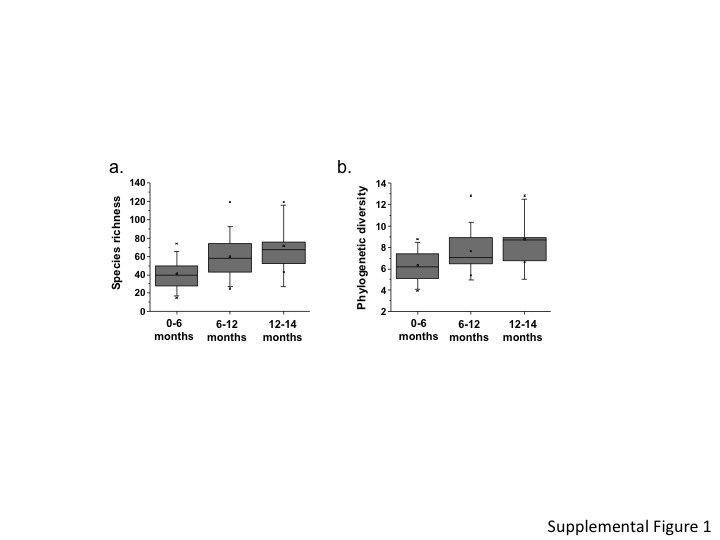

Supplement: Supplemental Figure 1 — Bacterial Phylogenetic Diversity (PD) and species richness (S) during the first 14 months of age increases over time. [file DataSheet1.ZIP › Supplemental Data/Supplemental Figure 1.JPEG]

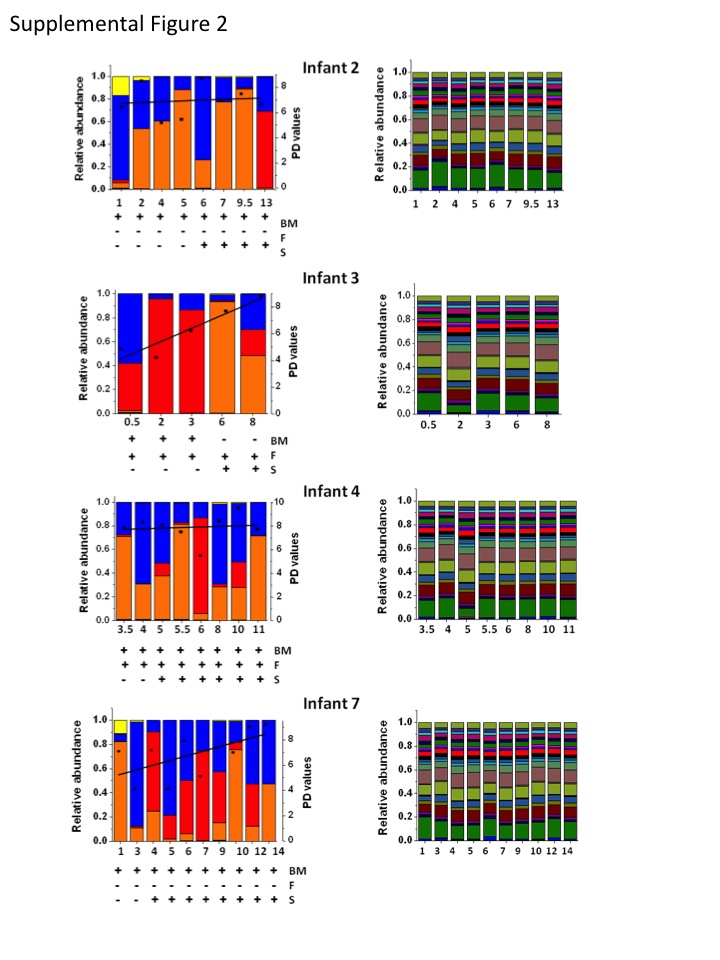

Supplement: Supplemental Figure 1 — Bacterial Phylogenetic Diversity (PD) and species richness (S) during the first 14 months of age increases over time. [file DataSheet1.ZIP › Supplemental Data/Supplemental Figure 2a.JPEG]

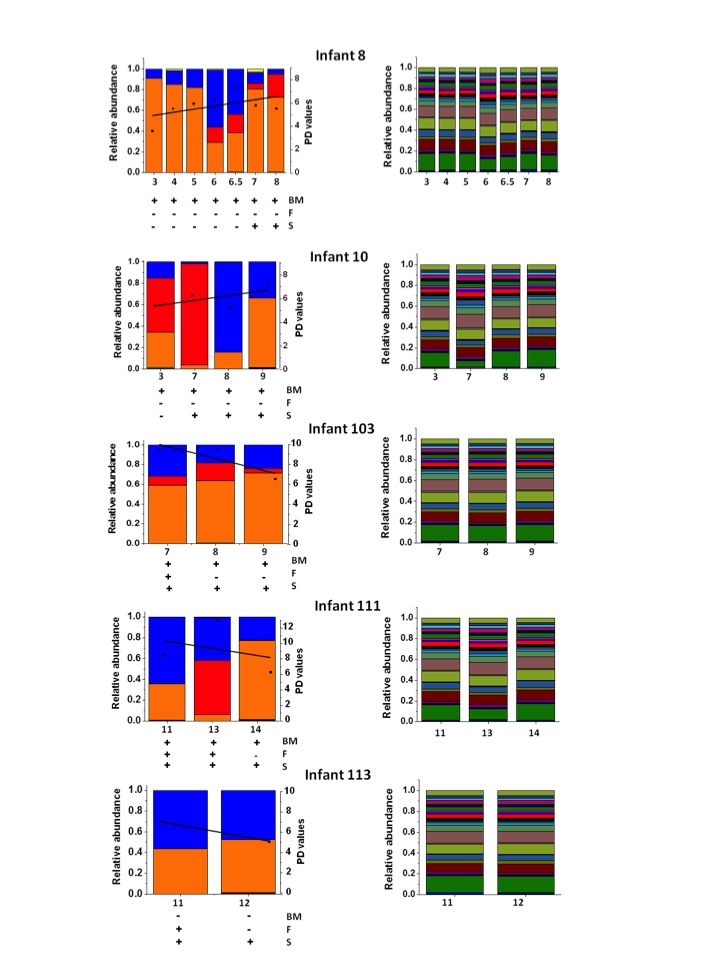

Supplement: Supplemental Figure 1 — Bacterial Phylogenetic Diversity (PD) and species richness (S) during the first 14 months of age increases over time. [file DataSheet1.ZIP › Supplemental Data/Supplemental Figure 2b.JPEG]

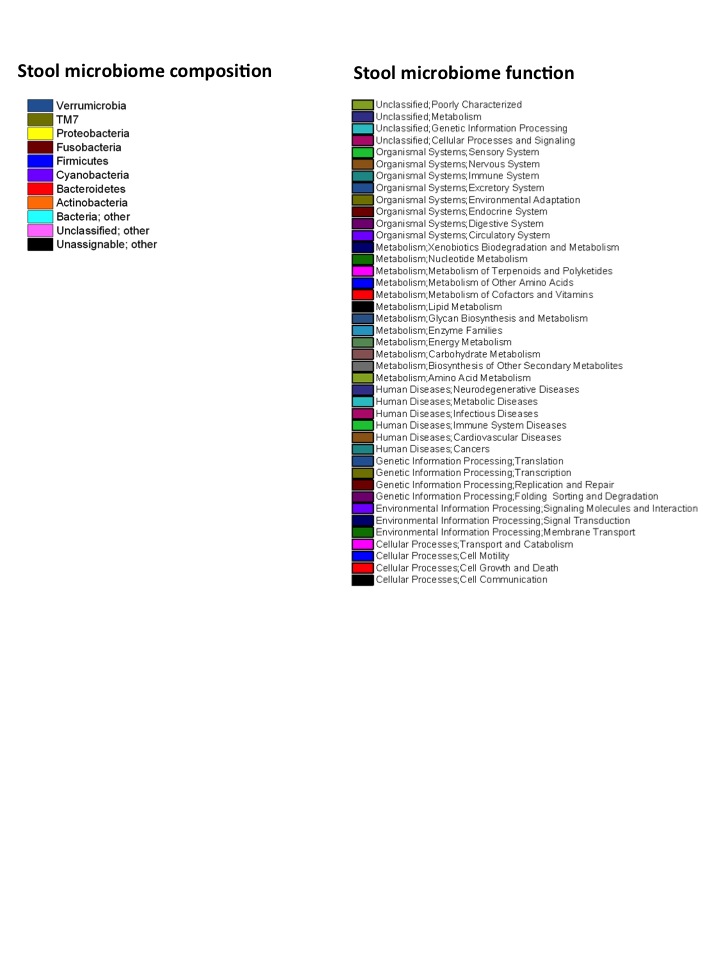

Supplement: Supplemental Figure 1 — Bacterial Phylogenetic Diversity (PD) and species richness (S) during the first 14 months of age increases over time. [file DataSheet1.ZIP › Supplemental Data/Supplemental Figure 2c.JPEG]

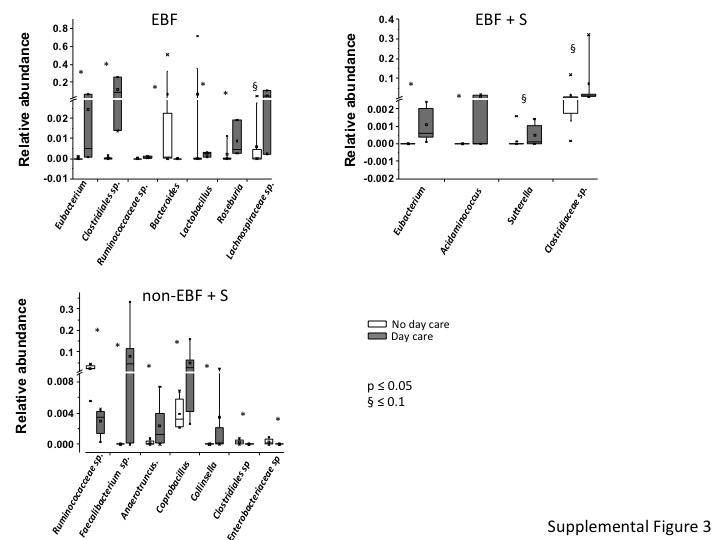

Supplement: Supplemental Figure 1 — Bacterial Phylogenetic Diversity (PD) and species richness (S) during the first 14 months of age increases over time. [file DataSheet1.ZIP › Supplemental Data/Supplemental Figure 3.JPEG]
